# Baicalin administration attenuates hyperglycemia-induced malformation of cardiovascular system

**DOI:** 10.1038/s41419-018-0318-2

**Published:** 2018-02-14

**Authors:** Guang Wang, Jianxin Liang, Lin-rui Gao, Zhen-peng Si, Xiao-tan Zhang, Guo Liang, Yu Yan, Ke Li, Xin Cheng, Yongping Bao, Manli Chuai, Li-guo Chen, Da-xiang Lu, Xuesong Yang

**Affiliations:** 1 Division of Histology & Embryology, Joint Laboratory for Embryonic Development & Prenatal Medicine, Medical College, Jinan University, Guangzhou, 510632 China; 2 Chinese Medicine College, Jinan University, Guangzhou, 510632 China; 3 Key Laboratory for Regenerative Medicine of the Ministry of Education, Jinan University, Guangzhou, 510632 China; 4 Department of Pediatrics and Neonatology, Institute of Fetal-Preterm Labor Medicine; The First Affiliated Hospital, Jinan University, Guangzhou, 510630 China; 5 Norwich Medical School, University of East Anglia, Norwich, Norfolk UK; 6 Division of Cell and Developmental Biology, University of Dundee, Dundee, DD1 5EH UK; 7 Department of Pathophysiology, Institute of Brain Research, Medical College, Jinan University, Guangzhou, 510632 China

## Abstract

In this study, the effects of Baicalin on the hyperglycemia-induced cardiovascular malformation during embryo development were investigated. Using early chick embryos, an optimal concentration of Baicalin (6 μM) was identified which could prevent hyperglycemia-induced cardiovascular malformation of embryos. Hyperglycemia-enhanced cell apoptosis was reduced in embryos and HUVECs in the presence of Baicalin. Hyperglycemia-induced excessive ROS production was inhibited when Baicalin was administered. Analyses of SOD, GSH-Px, MQAE and GABAA suggested Baicalin plays an antioxidant role in chick embryos possibly through suppression of outwardly rectifying Cl(−) in the high-glucose microenvironment. In addition, hyperglycemia-enhanced autophagy fell in the presence of Baicalin, through affecting the ubiquitin of p62 and accelerating autophagy flux. Both Baicalin and Vitamin C could decrease apoptosis, but CQ did not, suggesting autophagy to be a protective function on the cell survival. In mice, Baicalin reduced the elevated blood glucose level caused by streptozotocin (STZ). Taken together, these data suggest that hyperglycemia-induced embryonic cardiovascular malformation can be attenuated by Baicalin administration through suppressing the excessive production of ROS and autophagy. Baicalin could be a potential candidate drug for women suffering from gestational diabetes mellitus.

## Introduction

Roots of *Scutellaria baicalensis* are used in Chinese medicine and it reputedly calms fetuses in pregnant women^1,2^. Baicalin, a flavone glycoside (the glucuronide of Baicalein), is formed via the binding of glucuronic acid with Baicalein, in the roots of *Scutellaria baicalensis* and *Scutellaria lateriflora*. Baicalin is principally used in Asian countries as a herbal supplement because of its wide variety of health benefits, including anti-neuroinflammatory^3^, anti-cancer^4^ and anti-anxiety effects^5^. It can also improve lung function^6^ and fertility^7^.The most common mechanism of Baicalin being against pathogenesis is its protective effect by reducing the production of oxidative stress^8^. Interestingly, Qi et al.^9^ reported that Baicalin could enhance the developmental competence of mouse embryos through suppressing cellular apoptosis and HSP70 expression, and activating DNA methylation. However, whether or not Baicalin can prevent malformation of the cardiovascular system caused by environmental hazards at an early stage remains unclear. This study focuses on the potential beneficial effects of Baicalin on the early development of heart tube and vasculature, in order to explore whether or not Baicalin could be a potential candidate drug for pregnant women.

During embryonic development, the cardiovascular system appears first, since oxygen and nutrients need to be delivered to the tissues and waste products must be removed through blood circulation as the embryo develops. The cardiovascular system is composed of heart and blood vessels, including arteries, veins and capillaries in the adult. Heart and vasculature derive from both the embryonic mesoderm and extra-embryonic yolk sac. During early embryonic development, the heart initially forms in embryonic disc as a simple paired heart tube inside the forming pericardial cavity. During the process, the heart formation undergoes a series of transformations. A straight heart tube derives from the fusion of bilateral cardiomyocytes in primary/secondary heart fields at embryonic midline^10^, and then followed by the right looping of the heart tube and sepetation^11^. Endoderm-derived signals such as bone morphogenetic protein (BMP), fibroblast growth factor and Wnt antagonist are indispensable for precardiac mesoderm cells to differentiate into mature cardiomyocytes during cardiomyogenesis^12–14^. Moreover, Nkx2.5 and GATA factors are all considered to be the important cardiogenic transcription factors that characterize and induce cardiogenic differentiation^15^.

Meanwhile, vasculature formation occurs in embryogenesis at three different morphological stages, including vasculogenesis, angiogenesis and vascular remodeling. Both vasculogenesis and angiogenesis are accomplished in the prenatal period. Vasculogenesis depicts the formation of initial primitive vascular plexus derived from hemangioblasts in extra-embryonic yolk sacs or within the embryo. The earliest appearance is in blood islands located in the extra-embryonic region, in which the mesoderm contributes to blood islands and then these cells differentiate into both the epithelium of blood vessels and fetal red blood cells. Morphologically, these blood islands join together to form the primary blood plexus (vasculogenesis) that connects with the developing heart tube to form the integrated cardiovascular system. Angiogenesis involves the remodeling and expansion of the vascular plexus through endothelial sprouting and intussusceptive microvascular growth^16^. The yolk sac membrane (YSM) in the extra-embryonic region is the initial site where blood vessels and angioblasts develop, and the physiological function of YSM is to provide nutrition to the developing embryo. The chick chorioallantoic membrane (CAM) is a highly vascularized membrane underneath the inner surface of the eggshell, and CAM is formed by the fusion of the chorionic membrane and allantois during embryo development. Both chick YSM and CAM are excellent in vivo models for studying the angiogenesis because they can be easily accessed and manipulated in vitro^17–20^.

Gestational diabetes is due to glucose intolerance during pregnancy and presents as high blood glucose levels at the beginning of pregnancy. The incidence of congenital malformations in diabetic pregnancy has been reported to be 2−5 times higher than in non-diabetic pregnancy^21^. Dysplasia of the cardiovascular system during cardiogenesis and angiogenesis leads to constantly impaired fetal development or death. However, there is no effective drug for the protection of embryonic development of gestational diabetes mellitus. In our previous studies, early gastrulating chick embryo was employed as an experimental model in gestational diabetes-induced cardiovascular malformation^22,23^. We found both reactive oxygen species (ROS) and autophagy play an important role in chick embryo malformation. In the present study, Baicalin was shown to exhibit a protective effect on early cardiovascular development, and the underlying mechanisms were partly dissected using in vivo chick and mouse models, and HUVEC cell line.

## Materials and methods

### Mice and treatment

#### Reagents

Baicalin (99% purity) was purchased from Santa Cruz Biotechnology (Dallas, TX, USA). According to the results of Chen et al., 40 mg/kg body weight (BW) of Baicalin can alleviate the effect of mifepristone on the levels of serum estrogen/progesterone and the important molecules of canonical Wnt signaling pathway during peri-implantation period in mice^24^.

#### Experimental animal groups and treatments

Kunming mice were obtained from the Laboratory Animal Centre of Sun Yat-sen University (Guangzhou, China). Diabetes mellitus was induced in 8-week-old female mice by injecting streptozotocin (STZ; Sigma, St. Louis, MO, USA; dissolved in 0.01 mol/l citrate buffer, pH 4.5) at 75 mg/kg BW for three consecutive days. Blood glucose levels were measured by the Roche Accu-Chek Aviva Blood Glucose System (Roche, Penzberg, BY, Germany) for 7 days after STZ injection. Diabetes mellitus was characterized as non-fasting blood glucose level exceeding 16 mM^16,17^. Mice with diabetes mellitus were randomly divided into two groups: a 40 mg/kg BW Baicalin treatment group (*n* = 12) and a physiological saline treatment group (*n* = 11). In control (*n* = 6) and diabetes mellitus groups, the mice were administered with 0.1% dimethyl sulfoxide solution via an intra-gastric gavage for 1 week. The mice in Baicalin group were administered with 1 ml/day of Baicalin by intra-gastric gavage for 1 week.

This study was carried out in strict accordance with the recommendations of the Guide for the Care and Use of Laboratory Animals of the National Institutes of Health. The protocol was approved by the Committee on the Ethics of Animal Experiments of the Jinan University. All surgeries were performed under pentobarbital anesthesia, and all efforts were employed to minimize suffering.

### Avian embryos and treatment

Fertilized chick eggs were obtained from the Avian Farm of the South China Agriculture University. The eggs were incubated until the required stage^25^ in a humidified incubator (Yiheng Instrument, Shanghai, China) at 38 °C and 70% humidity. For early gastrula embryos, HH0 (Hamburger and Hamilton stage)^25^ chick embryos were prepared and incubated in the absence/presence of Baicalin (Sigma, USA) and/or high glucose (Sigma, USA) and Rapa (LC Labs, USA) using early chick culture (EC culture) as described previously^26^.

The embryos were harvested at the desired time based on the experimental requirements after incubation at 38 ^o^C. All of the embryos were photographed using a stereomicroscope (Olympus MVX10, Japan) before being fixed with 4% paraformaldehyde for morphological and gene expression analysis. Only the surviving embryos were used for further study. For the histological analysis, the treated embryos or yolk sacs were dehydrated, embedded in paraffin wax and serially sectioned at 5 µm using a microtome (Leica RM2126RT, Germany). The sections were de-waxed in xylene, rehydrated and stained with either hematoxylin and eosin dye or immunofluorescent stain, and photographed using a fluorescent microscope (Olympus IX50) with the NIS-Elements F3.2 software package.

### Assessment of angiogenesis on chick embryonic YSM

Briefly, the eggs were treated with simple saline, 100 μl of 3.75 mg/ml  2,2′-Azobis(2-methylpropionamidine) dihydrochloride (AAPH) or/and 6 μM Baicalin per egg^18^. The solutions were directly injected into the blunt air chamber of the fertilized egg and then the embryos were harvested after a further 4.5 days of incubation at 38 °C. All of the harvested YSMs were photographed using a stereomicroscope (Olympus MVX10, Tokyo, Japan) before being used for molecular analyses.

### Assessment of angiogenesis using chick CAM

As described previously^27^, chick embryos were incubated until day 9 when the CAM was well developed. The embryos were treated with 100 μl of simple saline, high glucose (50 mM) and/or Baicalin (6 μM) for 48 h and all surviving embryos were then harvested for analysis. The CAM and accompanying blood vessels in the control and high glucose (50 mM) and/or Baicalin (6 μM)-treated embryos were photographed using a Canon Powershot SX130 IS digital camera (12.1 M Pixels).

### Cell lines and culture

Human umbilical vascular endothelial cells (HUVECs), a gift from Zhi Huang’s laboratory, were cultured in Dulbecco’s modified Eagle’s medium (DMEM) (Gibco, Shanghai, China) supplemented with 10% fetal bovine serum (FBS), and incubated at 37 °C and 5% CO_2_.

### Histology

Briefly, 12-week-old mouse livers, mouse kidney (control, STZ-induced-diabetes mellitus, and STZ-induced-diabetes mellitus with Baicalin treatment) were fixed in 4% paraformaldehyde at 4 °C for 24 h. The specimens were then dehydrated, cleared in xylene and embedded in paraffin wax. The embedded specimens were serially sectioned at 5 μm using a rotary microtome (Leica, RM2126RT, Wetzlar, Hessen, Germany). The sections were stained with hematoxylin and eosin (H&E), periodic acid Schiff (PAS) reaction, Masson’s trichrome dyes (Masson staining), Sirius red or immunohistochemically. The Masson and Sirius red stains were used to reveal the presence of fibrosis in liver sections and the histological alterations of mice’s kidney. Photographs were captured of the stained histological sections using an epifluorescence microscope (Olympus IX51, Leica DM 4000B).

### Immunofluorescent staining and F-actin/Hoechst/PI staining

Chick embryos were harvested after a given time incubation and fixed in 4% PFA overnight at 4 °C. Whole-mount embryo immunostaining was performed using the following antibody: MF-20 (1:500, DSHB, USA). Briefly, the fixed embryos were then incubated with this primary antibody at 4 °C overnight on a shaker. Following extensive washing, the embryos were incubated with anti-rabbit IgG conjugated to Alexa Fluor 488 overnight at 4 °C on a rocker. For F-actin detection, the cultured cells were stained using phalloidin-Alexa-Fluor 488 (1:200, Invitrogen, USA) at room temperature for 2 h. All the embryos were later counterstained with 4',6-diamidino-2-phenylindole (DAPI; 1:1000, Invitrogen, USA) at room temperature for 1 h. For Hoechst (1:1000, Sigma, USA)/propidium iodide (PI, 1:1000, Sigma, USA) staining, the cells were cultured and washed twice with cold phosphate-buffered saline (PBS), and then incubated with Hoechst/PI for 45 min at 37 °C in the dark.

### Dihydroethidium and CCK8 assays

Dihydroethidium (DHE) staining was performed at the end of cell culture using DHE fluorescent probe (Beyotime, Shanghai, China) to detect the presence of superoxide anion (O_2_^−^). The cells were incubated with 10 μM DHE for 30 min, at 37 °C, and then collected for analyses according to the manufacturer’s instructions. HUVECs (control, 6 μM Baicalin, 50 mM glucose and 6 μM Baicalin+50 mM glucose group) were seeded into 96-well plates. These cells (1 × 10^6^ cells/ml) were maintained in DMEM+10% fetal bovine serum at 37 °C and 5% CO_2_. The cell viability was assessed using CCK8 assay (cholecystokinin-8). Briefly, 10 μl of CCK8 reagent (Dojindo, Kumamoto, Japan) was added to the 96-well plates and incubated continually for 6, 12, 24 and 48 h at 37 °C. The absorbance values were measured at 450 nm using a Bio-Rad model 450 microplate reader (Bio-Rad, Hercules, CA, USA). The cell viability was indirectly determined by examining the ratio of the absorbance value of Baicalin or/and glucose-treated cells relative to the control cells, from this experiments.

### Western blot

Chick embryos (HH10) were collected and lysed with CytoBuster™ Protein Extraction Reagent (#71009, Novagen). The total protein concentration was determined using a BCA quantification kit (BCA01, DingGuo BioTECH, CHN). Samples containing equal amounts of protein were resolved by odium dodecyl sulfate–polyacrylamide gel electrophoresis and then transferred to polyvinylidene difluoride membranes (Bio-Rad). The membranes were blocked with 5% Difco™ skimmed milk (BD) and then incubated with primary and secondary antibodies. The antibodies used were GATA4 (Bioworld, USA); LC3B (Cell Signaling Technology, USA); Beclin1 (Bioworld, USA); C-Caspase-3 (Cell Signaling Technology, USA); p62 (Sigma, USA); β-actin (Proteintech, USA); horseradish peroxidase-conjugated anti-mouse IgG and anti-rabbit IgG (Cell Signaling Technology, USA). All primary and secondary antibodies used were diluted to 1:1000 and 1:2000 in 5% skimmed milk, respectively. The protein bands of interest were visualized using an ECL kit (#34079, Thermo Fischer Scientific Inc., USA) and GeneGnome5 (Syngene, UK). The staining intensity of the bands was determined and analyzed using Quantity One software (Bio-Rad).

### In situ hybridization

Whole-mount in situ hybridization of chick embryos was performed according to a standard in situ hybridization protocol^28^. Digoxigenin-labeled probes were synthesized against VE-Cadherin^21^. The whole-mount stained embryos were photographed by a stereomicroscope (Olympus MVX10, Tokyo, Japan).

### RNA isolation and reverse transcription-PCR (RT-PCR)

Total RNA was isolated from HH10 chick embryos using a Trizol kit (Invitrogen, USA) according to the manufacturer’s instructions. First-strand complementary DNA (cDNA) was synthesized to a final volume of 25 μl using SuperScript RIII first-strand (Invitrogen, USA). Following reverse transcription, PCR amplification of the cDNA was performed as described previously^29,30^. The sets of primers used for RT-PCR are described in the Supplementary Fig. 1. The PCR reactions were performed in a Bio-Rad S1000TM Thermal cycler (Bio-Rad, USA). The final reaction volume was 50 μl, comprising 1 μl of first-strand cDNA, 25 μM forward primer, 25 μM reverse primer, 10 μl PrimeSTARTM Buffer (Mg2+ plus), 4 μl dNTP Mixture (TaKaRa, Japan), 0.5 μl PrimeSTARTM HS DNA Polymerase (2.5 U/μl TaKaRa, Japan) and RNase-free water. cDNA was amplified for 30 cycles. One round of amplification was performed at 94 °C for 30 s and then 30 s at 58 °C and 30 s at 72 °C. The PCR products (20 μl) were resolved using 1% agarose gels (Biowest, Spain) in 1× TAE buffer (0.04 M Trisacetate and 0.001 M EDTA) and 10,000× GeneGreen Nucleic Acid Dye (TIANGEN, China) solution. The resolved products were visualized using a transilluminator (SYNGENE, UK), and photographs captured using a computer-assisted gel documentation system (SYNGENE). Each of these experiments was replicated at least three times.

### RNA isolation and quantitative PCR

Total RNA was isolated from chick embryo (HH7 and HH10) HUVECs using a Trizol kit (Invitrogen, USA) according to the manufacturer’s instructions. First-strand cDNA was synthesized to a final volume of 20 μl using iScriptTM cDNA Synthesis Kit (Bio-Rad, USA). Following reverse transcription, PCR amplification of the cDNA was performed as described previously^29,30^. SYBR® Green qPCR assays were then performed using a PrimeScriptTM RT reagent kit (Takara, Japan). All specific primers used are described in Supplementary Fig. 2. Reverse transcription and amplification reactions were performed in Bio-Rad S1000TM (Bio-Rad, USA) and ABI 7000 thermal cyclers, respectively. The housekeeping gene *PPIA* was run in parallel to confirm that equal amounts of RNA were used in each reaction. The ratio between the intensities of the fluorescently stained bands corresponding to genes and PPIA was calculated to quantify the level of the transcripts for those gene mRNAs.

### Quantitation of apoptotic cells

Annexin V-FITC (BD Bioscience, USA) and PI double staining were used to identify and quantify apoptotic cells present in the HUVECs (control, 50 mM glucose, 6 μM Baicalin, 6 μM Baicalin+50 mM glucose, 40 μg/ml Vitamin C+ 50mM glucose, 10 μM chloroquine+50 mM glucose treated for 48 h). Briefly, the cells were collected and resuspended in cold PBS at a density of 1 × 10^6^ cells/ml and then introduced into 200 μl of the Annexin V-binding buffer. The samples were then incubated with 2 μl fluorescein isothiocyanate (FITC)-labeled Annexin V and 2 μl PI at room temperature for 15 min and immediately analyzed by a FACS Calibur flow cytometer (BD, NJ, USA). The acquired data were evaluated using FCS-Express software version 3.0 (De Novo).

### Measurement of oxidative stress

Intracellular ROS was determined using a non-fluorescent dye DCF-DA (2′,7′-dichlorodihydrofluorescein diacetate) (Sigma, USA), which is oxidized by ROS to the fluorescent dye DCF (2′,7′-dichlorofluorescin). The control and high glucose (50 mM) and/or Baicalin (6 μM)-treated HUVECs were incubated in 10 μM DCF-DA for 20 min. Fluorescence was measured with a BD FACSAria (USA).

Superoxide dismutase (SOD), glutathione peroxidase (GSH-Px) and malonicdialdehyde (MDA) are expressed in the HH10 chick embryos. SOD, GSH-Px and MDA activities were elucidated using commercial kits (Nanjing, China). The results from control and the three treated groups were analyzed based on the differences of their respective fluorescein decay curves.

### Detection of intracellular chloride ion

Intracellular chloride ion concentration was measured using *N*-[ethoxycar bonylmethyl]-6-methoxy-qu inolinium bromide (MQAE) (Beyotime, China), a fluorescent chloride ion indicator. Upon binding halide ions such as chloride, MQAE is quenched, resulting in a decrease in fluorescence without a shift in wavelength. HUVECs were incubated in Krebs-HEPES buffer (PanEra, China) containing 5 mM MQAE at room temperature for 30 min, and then washed three times with Krebs-HEPES buffer. MQAE-loaded cells (over 1 × 10^5^ cells) were then mounted on a glass coverslip for 20 min at room temperature.

### Data analysis

The BVD in the CAM, YSM or blood island density was analyzed using the Image Pro-Plus 5.0 software^20^. Statistical analyses for all the experimental data generated were performed using a SPSS 13.0 statistical package program for Windows. The data were presented as mean ± SD. Statistical significance were determined using paired Test, independent samples *T*-test or one-way analysis of variance. *P* < 0.05 was considered to be significant.

## Results

### Screening the optimal concentration of Baicalin for administration to early chick embryos

**Fig. 1:**
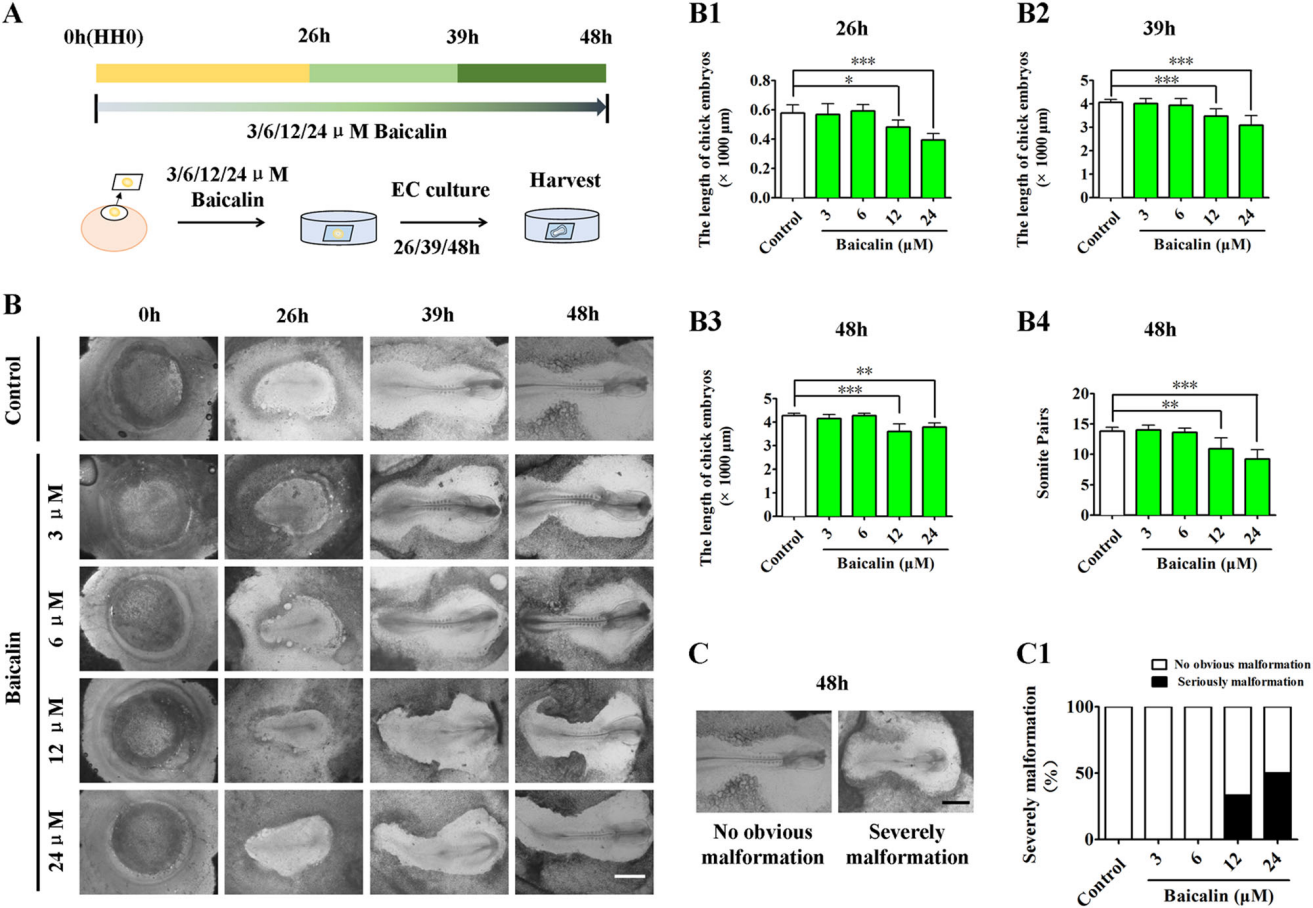
The assessment of gastrula chick embryo development in the absence/presence of various concentrations of Baicalin. **a** The sketches illustrating the EC culture of HH0 gastrula chick embryos in the absence/presence of Baicalin (see Materials and method section for details). **b** The representative bright-field images were taken from control (first row), 3 μM Baicalin (second row), 6 μM Baicalin (third row), 12 μM Baicalin (fourth row) and 24 μM Baicalin (fifth row) groups at incubation times of 0, 26, 39 and 48 h, respectively. **b1–b4** Bar charts showing the comparison of chick embryo length at 26 h (**b1**), 39 h (**b2**), 48 h (**b3**) and somite pair numbers at 48 h (**b4**) in the absence/presence of various concentrations of Baicalin. **c** The representative bright-field images of normal (left) and abnormal (right) gastrula chick embryos after 48 h of incubation. **c1** Bar chart showing the comparison of normal and abnormal chick embryo numbers in the absence/presence of various concentrations of Baicalin.*p<0.05 compared with control group; **p<0.01 compared with control group; ***p<0.001 compared with control group. Scale bars = 1000 µm in (**b**, **c**)

To determine whether or not there is a cytotoxic effect on developing embryos following the application of Baicalin^31^, HH0 chick embryos were first incubated with 3, 6, 12 and 24 μM Baicalin for 26, 39 and 48 h in EC culture (Fig. 1a), respectively. The developing chick embryos treated with sample saline (control) or various concentrations (3, 6, 12 and 24 μM) of Baicalin were photographed at incubation times of 0, 26, 39 and 48 h (Fig. 1b). Using embryo length and somite pair number as the embryonic developmental indices, 3 and 6 μM Baicalin showed no effect, but exposure to 12 and 24 μM Baicalin significantly reduced length development (*p* < 0.05, *p* < 0.01, *p *< 0.001; *n* > 6 embryos in each group) and somite pair numbers in comparison to control embryos (*p* < 0.01, *p* < 0.001; *n* = 10; Fig. 1b1-b4). Meanwhile, it was observed that 3 μM and 6 μM Baicalin had no obvious effect on embryonic malformation, but higher exposures of 12 and 24 μM had a significant effect (severe malformation: 33% in 12 μM and 50% 24 μM, *n* > 6 embryos in each group; Fig. 1c-c1). Therefore, concentrations of 3 and 6 μM were used in subsequent experiments.

### Baicalin administration successfully reversed hyperglycemia-inhibited development of early chick embryos

**Fig. 2:**
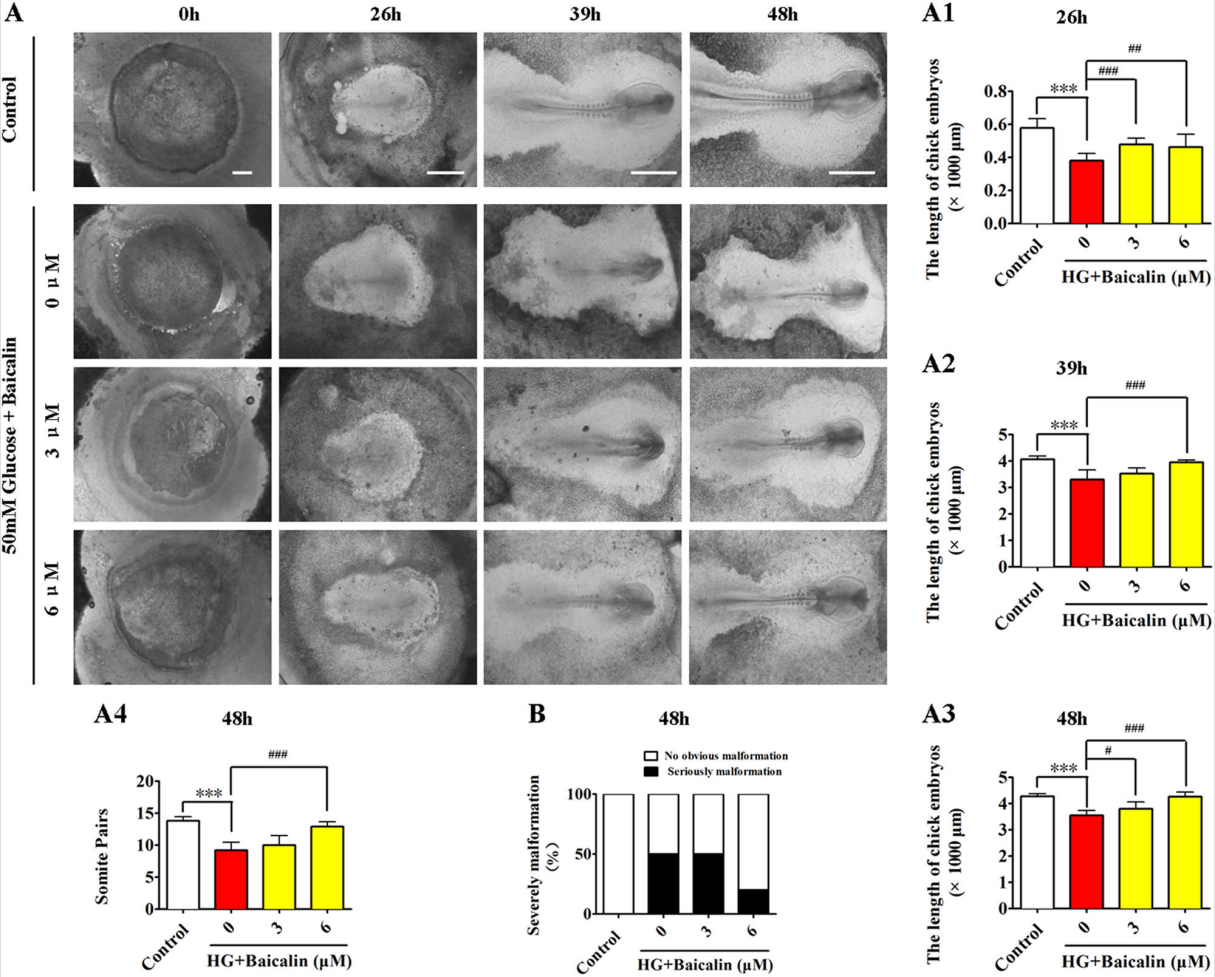
The assessment of gastrula chick embryo development in the absence/presence of HG and Baicalin. **a** The representative bright-field images were taken from control (first row), 50 mM glucose + 0 μM Baicalin (second row), 50 mM glucose+3 μM Baicalin (third row) and 50 mM glucose+6 μM Baicalin (fourth row) groups at incubation times of 0, 26, 39 and 48 h, respectively. **a1**–**a4**Bar charts showing the comparison of chick embryo length at 26 h (**a1**), 39 h (**a2**), 48 h (**a3**) and somite pair numbers at 48 h (**a4**) in the absence/presence of 50 mM glucose+various concentrations of Baicalin. **b** Bar chart showing the comparison of severely abnormal chick embryo percentages in the absence/presence of various concentrations of Baicalin. HG high glucose. ***p<0.001 compared with control group; ^#^p<0.05 compared with HG group; ^##^p<0.01 compared with HG group; ^###^p<0.001 compared with HG group. Scale bars = 1000 µm in (**a**)

Hyperglycemia has been shown to negatively affect embryo development^32^. Here, through comparing the development of gastrula chick embryos exposed to 50 mM glucose (high glucose (HG)) and/or 3 or 6 μM Baicalin for 26, 39 and 48 h (Fig. 2a), it was discovered that HG dramatically inhibited the length and somite pair numbers of chick embryos (*p* < 0.001; *n* > 6 embryos in each group). However, the addition of 6 μM Baicalin could significantly reverse the inhibitive effect on embryo development in 48 h (*p* < 0.001 compared with the HG group; *n* > 6 embryos in each group; Fig. 2a1-b). Simultaneously, the numbers of malformed embryos were also reduced to some degree after addition of 6 μM Baicalin (severe malformation: 0% in control, 50% in HG, 50% in HG+3 μM Baicalin and 20% in HG+6 μM Baicalin, *n* > 6 embryos in each group, Fig. 2b). This indicates that Baicalin administration exerts a positive effect on hyperglycemia-inhibited embryo development.

### Baicalin administration saved hyperglycemia-induced malformation of early chick embryonic cardiovascular system

**Fig. 3:**
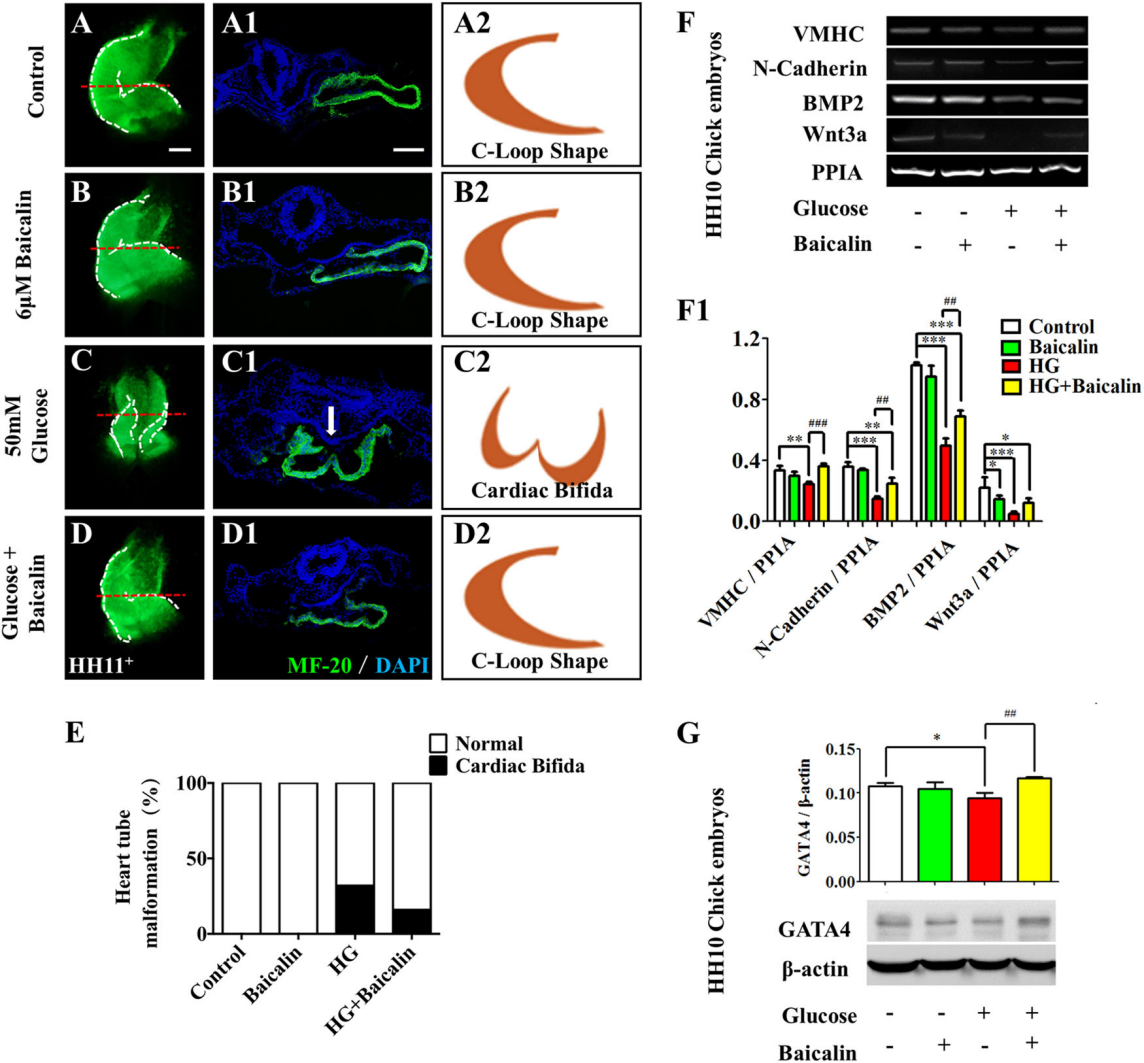
The assessment of chick heart tube formation in the absence/presence of HG and Baicalin. **a**–**d** The representative MF-20 immunofluorescent staining of chick heart tubes from control (**a**), 6 μM Baicalin (**b**), 50 mM glucose (**c**) and 6 μM Baicalin+50 mM glucose (**d**) group. **a1**–**d1** The transverse sections were taken from the levels indicated by dotted lines in (**a**–**d**), respectively. **a2**–**d2** The sketches illustrating the shapes of heart tubes at transverse sections in **a1**–**d1** respectively. **e** Bar chart showing the comparison of the incidences in the absence/presence of HG or/and Baicalin. **f**–**f1** The RT-PCR data showing the expressions of VMHC, N-Cadherin, BMP2 and Wnt3a in HH10 chick embryos exposed to either simple saline (control) or HG or/and Baicalin (**f**), which was quantitatively analyzed in (**f1**). **g** The western blot data showing the GATA4 expression in HH10 chick embryos exposed either simple saline (control) or HG or/and Baicalin (**g**), which was quantitatively analyzed in (**g1**). *p<0.05 compared with control group; **p<0.01 compared with control group; ***p<0.001 compared with control group; ^##^p<0.01 compared with HG group; ^###^p<0.001 compared with HG group. Scale bars = 1000 µm in (**a**–**d**) and (**a1**–**d1**)

Previous studies have shown that hyperglycemia could enhance the risk of heart tube malformation during embryogenesis^22^. MF-20 immunofluorescent staining showed that hyperglycemia induced a high incidence of cardiac bifida (32%, Fig. 3c-c2,e) which was clearly suppressed by addition of 6 μM Baicalin (16%, Fig. 3d-d2,e). Normal C-loop heart tubes could only be observed in the control and 6 μM Baicalin groups (100%, *n* = 25 in each group, Fig. 3a-a2,b-b2,e). The RT-PCR data showed that the expressions of heart tube formation-related genes, including VMHC, N-Cadherin, Wnt3a and BMP2, were down-regulated in the presence of HG (*p* < 0.01, *p* < 0.001), but VMHC, N-Cadherin, Wnt3a and BMP2 recovered to an extent following addition of 6 μM Baicalin (*p* < 0.01, *p* < 0.001 compared to the HG group; Fig. 3f-f1). A similar tendency was observed in the expression of GATA4 as indicated by western blot data (*p* < 0.05 compared with the control group, *p* < 0.01 compared with the HG group, Fig. 3g). Most of these genes were not changed significantly in the Baicalin group compared with the control group (*p* > 0.05).

**Fig. 4:**
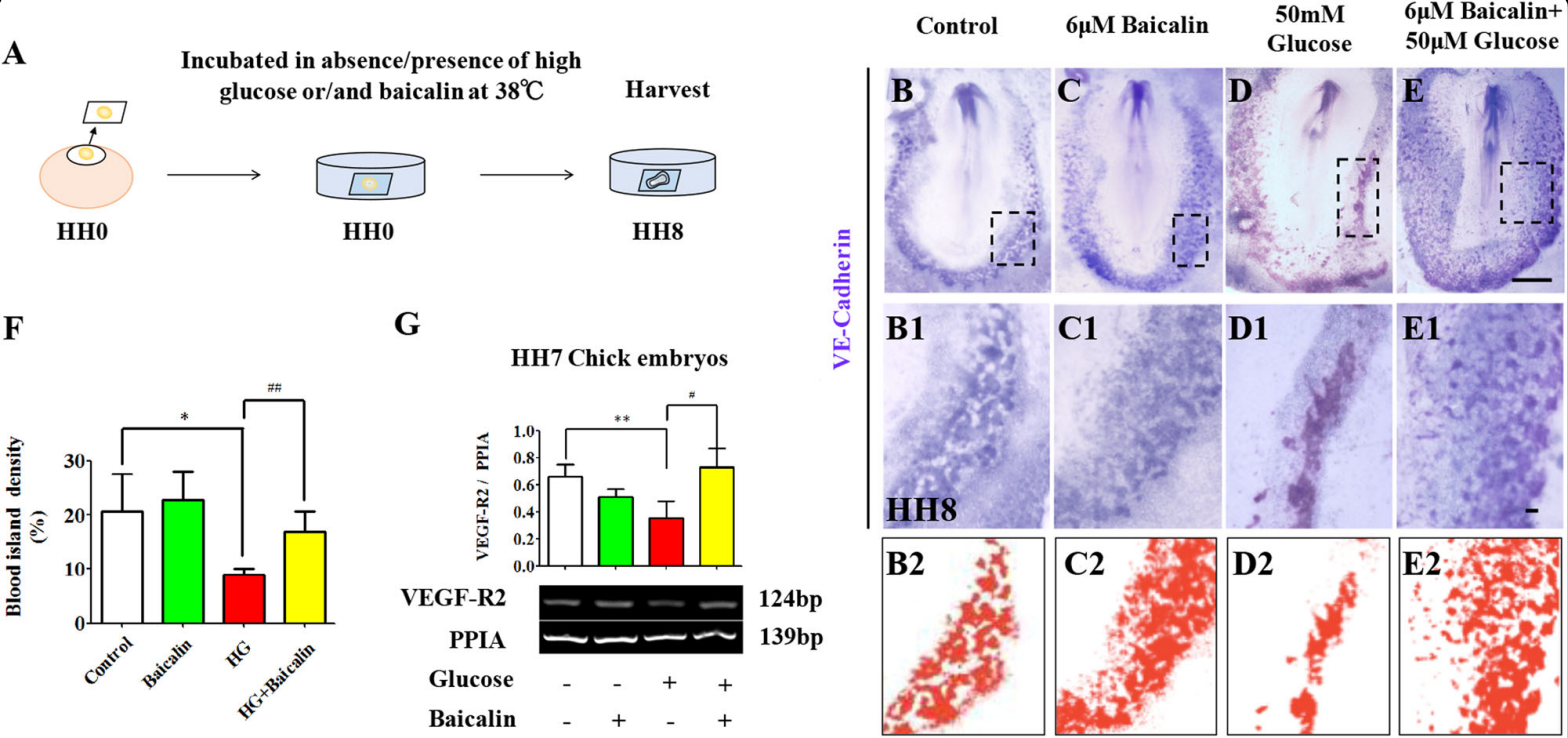
The assessment of blood island formation in the absence/presence of HG and Baicalin. **a** The sketches illustrating the EC culture of HH0 gastrula chick embryos in the absence/presence of HG or/and Baicalin. **b**–**e** Whole-mount in situ hybridization of VE-Cadherin was implemented on the HH8 chick embryos exposed to sample saline (control) (**b**), 6 μM Baicalin (**c**), 50 mM glucose (**d**) and 6 μM Baicalin+50 mM glucose (**e**). **b1**–**e1** The high magnification images were taken from the sites indicated by dotted squares in (**b1**–**e1**), respectively. **b2**–**e2** The sketches illustrating the shapes of blood islands in (**b1**–**e1**), respectively. **f** Bar chart showing the comparison of the blood island density on chick extra-embryonic regions in absence/presence of HG or/and Baicalin. **g**The RT-PCR data showing the expressions of VEGF-R2 in HH7 chick embryos exposed to either simple saline (control) or HG or/and Baicalin. *p<0.05 compared with control group; **p<0.01 compared with control group; ^#^p<0.05 compared with HG group; ^##^p<0.01 compared with HG group. Scale bars = 1000 µm in (**b**–**e**) and 100 µm in (**b1**–**e1**)

Vasculogenesis starts from blood islands expressed in VE-Cadherin, the blood island marker. VE-Cadherin in situ hybridization in whole-mount HH8 chick embryos (Fig. 4a) showed that blood island formation was not affected by 6 μM Baicalin (*p* > 0.05, Fig. 4b-c,b1-c1,b2-c2,f), was suppressed by 50 mM glucose (*p* < 0.001), but the reduced blood islands increased again when Baicalin and glucose were applied together (*p* < 0.01 compared with HG group, *n* = 8 in each group, Fig. 4d-e,d1-e1,d2-e2,f). This suggests that Baicalin administration could rescue the hyperglycemia-inhibited vasculogenesis to some extent.

Furthermore, whether or not Baicalin administration could rescue HG-inhibited angiogenesis^23^ was studied using the chick CAM model, illustrated in Fig. S2A. Chick embryo weight on the harvest day (day 9) was increased slightly when exposed to 6 μM Baicalin (*p* > 0.05), and reduced when exposed to 50 mM glucose (*p* < 0.05), but this reduction was reversed after exposure to 6 μM Baicalin (*p* < 0.01 compared with HG group, *n* > 10 embryos in each group; Fig. S2B). Compared to the angiogenesis on CAM in control group (*p* < 0.05, Fig. S2C), it was clear that 6 μM Baicalin alone did not change the blood vessel density (*p* > 0.05, Fig. S2D), and that 50 mM glucose dramatically inhibited angiogenesis (*p* < 0.05, Fig. S2E). The combination of 6 μM Baicalin and 50 mM glucose significantly reversed the reduction of blood vessel density induced by HG (*p* < 0.05 compared with HG group, *n* > 8 in each group; Fig. S2F). This is quantitatively analyzed in Fig. S2G.

In addition, the RT-PCR data showed that expressions of vasculogenesis-related genes VEGF-R2 were down-regulated in the presence of HG (*p* < 0.01), but there was a partial recovery after addition of 6 μM Baicalin (*p* < 0.05 compared with HG group, Fig. 3g).

### Baicalin administration rescued hyperglycemia-induced cell proliferative reduction and apoptosis increase

**Fig. 5:**
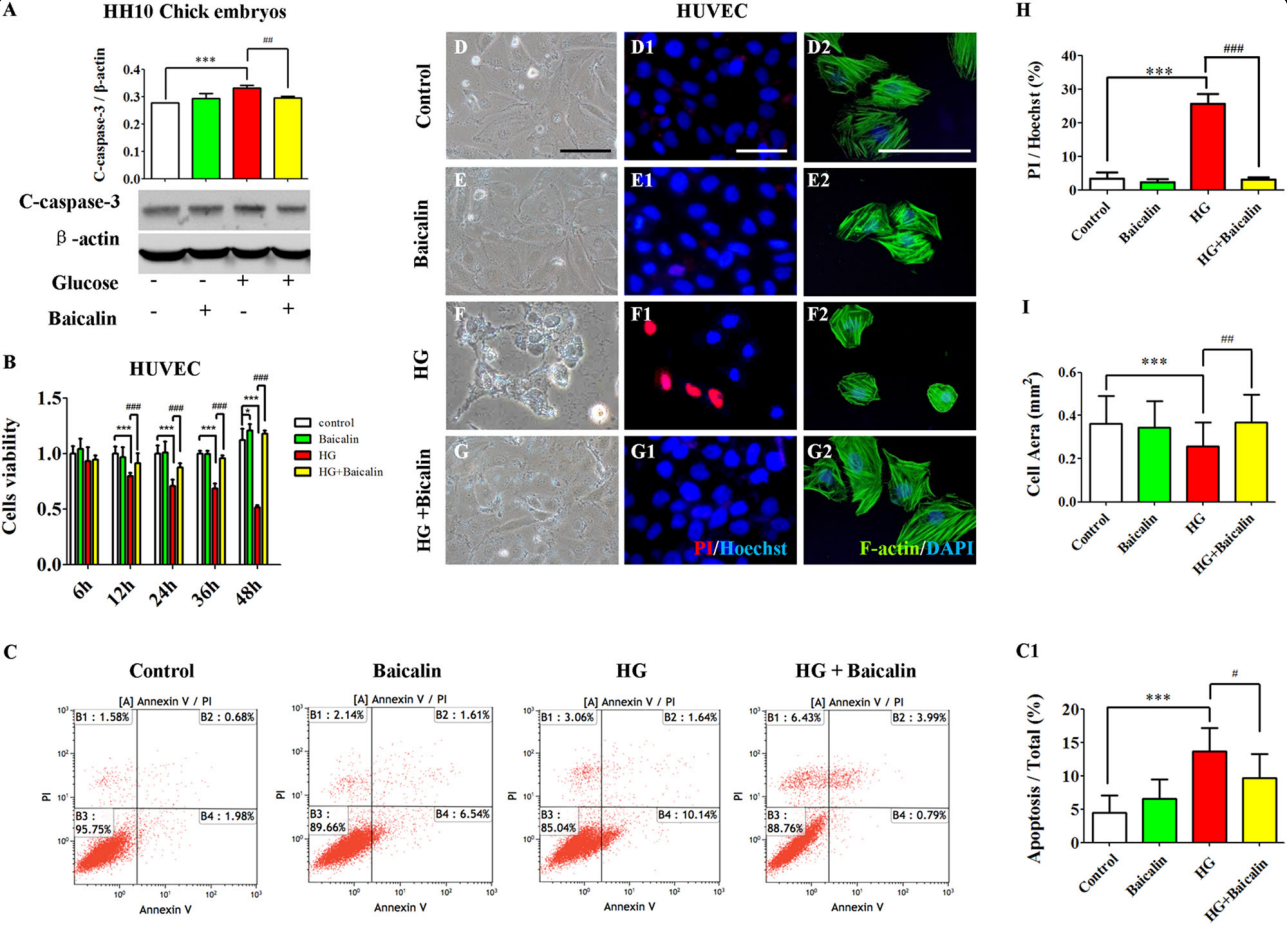
The extent of apoptosis in the absence/presence of HG and Baicalin. **a** The western blot data showing the C-caspase-3 expression in HH10 chick embryos exposed with either simple saline (control) or HG or/and Baicalin. **b** Bar chart showing the comparison of CCK8 values of HUVECs incubated with/without HG or/and Baicalin. **c** The propidium iodide (PI) flow cytometric assay was implemented in 48-h incubated HUVECs from control, 6 μM Baicalin, 50 mM glucose and 6 μM Baicalin+50 mM glucose group. **c1** The bar chart showing the comparison of the values of B2 (late apoptosis)+B4 (early apoptosis) in the cultured HUVECs among control, 6 μM Baicalin, 50 mM glucose and 6 μM Baicalin+50 mM glucose group. (**d**–**g**) The representative bright-field images of 48-h cultured HUVECs from control (**d**), 6 μM Baicalin (**e**), 50 mM glucose (**f**) and 6 μM Baicalin+50 mM glucose (**g**) group. **d1**–**g1** The fluorescent staining of PI and Hoechst was implemented on 48-h cultured HUVECs as in (**d**–**g**), respectively. **d2**–**g2** The fluorescent staining of F-actin and DAPI was implemented on 48-h cultured HUVECs as in (**d**–**g**), respectively. **h** Bar chart showing the cell surface area of HUVECs in the absence/presence of HG or/and Baicalin. **i** Bar chart showing the ratio comparison between PI^+^ and Hoechst^+^ HUVECs in the absence/presence of HG or/and Baicalin. *p<0.05 compared with control group; ***p<0.001 compared with control group; ^#^p<0.05 compared with HG group; ^##^p<0.01 compared with HG group; ^###^p<0.001 compared with HG group. Scale bars = 100 µm in (**d**–**g1**) and 50 µm in (**d2**–**g2**)

Western blot data showed that the expression of apoptosis-related gene C-caspase-3 increased in the presence of HG (*p* < 0.001), but was reversed to some extent by addition of 6 μM Baicalin (*p* < 0.01 compared with HG group, Fig. 5a) in early chick embryo. Furthermore, HUVECs were incubated with 6 μM Baicalin/50 mM glucose or in combination for 12, 24, 36 and 48 h. The cell counting kit-8 (CCK8) was then used to detect cell proliferation and viability (Fig. 5b). The results showed that the HUVEC proliferation and viability dropped in the presence of HG at 12, 24, 36 and 48 h of incubations (*p* < 0.001), but all of these reductions increased again when 6 μM Baicalin and 50 mM glucose (*p* < 0.001 was added together compared with HG group, *n* > 8 in each group; Fig. 5b). The data from flow cytometry showed that 50 mM glucose could significantly enhance HUVEC apoptosis (*p* < 0.001), and this increased apoptosis induced by HG was dramatically suppressed by the addition of 6 μM Baicalin (*p* < 0.05 compared with HG group, *n* = 3 in each group, Fig. 5c-c1). Furthermore, using PI fluorescent staining, it could be demonstrated that 50 mM glucose administration greatly increased the PI^+^ HUVEC numbers (*p* < 0.001), whereas HG-induced elevation of PI^+^ HUVEC numbers basically returned to normality (*p* < 0.001 compared with HG group, *n* = 3 in each group, Fig. 5d-h,d1-g1). Meanwhile, the HUVEC shape outlined by F-actin staining was observed to shrink in the presence of 50 mM glucose (*p *< 0.001, *n* > 18 in each group, Fig. 5d-h,d2-g2), and then it generally kept in size when additional 6 μM Baicalin was added (*p* < 0.001 compared to the HG group, *n* > 18 in each group, Fig. 5i).

### Baicalin administration stabilized the hyperglycemia-induced oxidative stresses’ secondary effect on angiogenesis

**Fig. 6:**
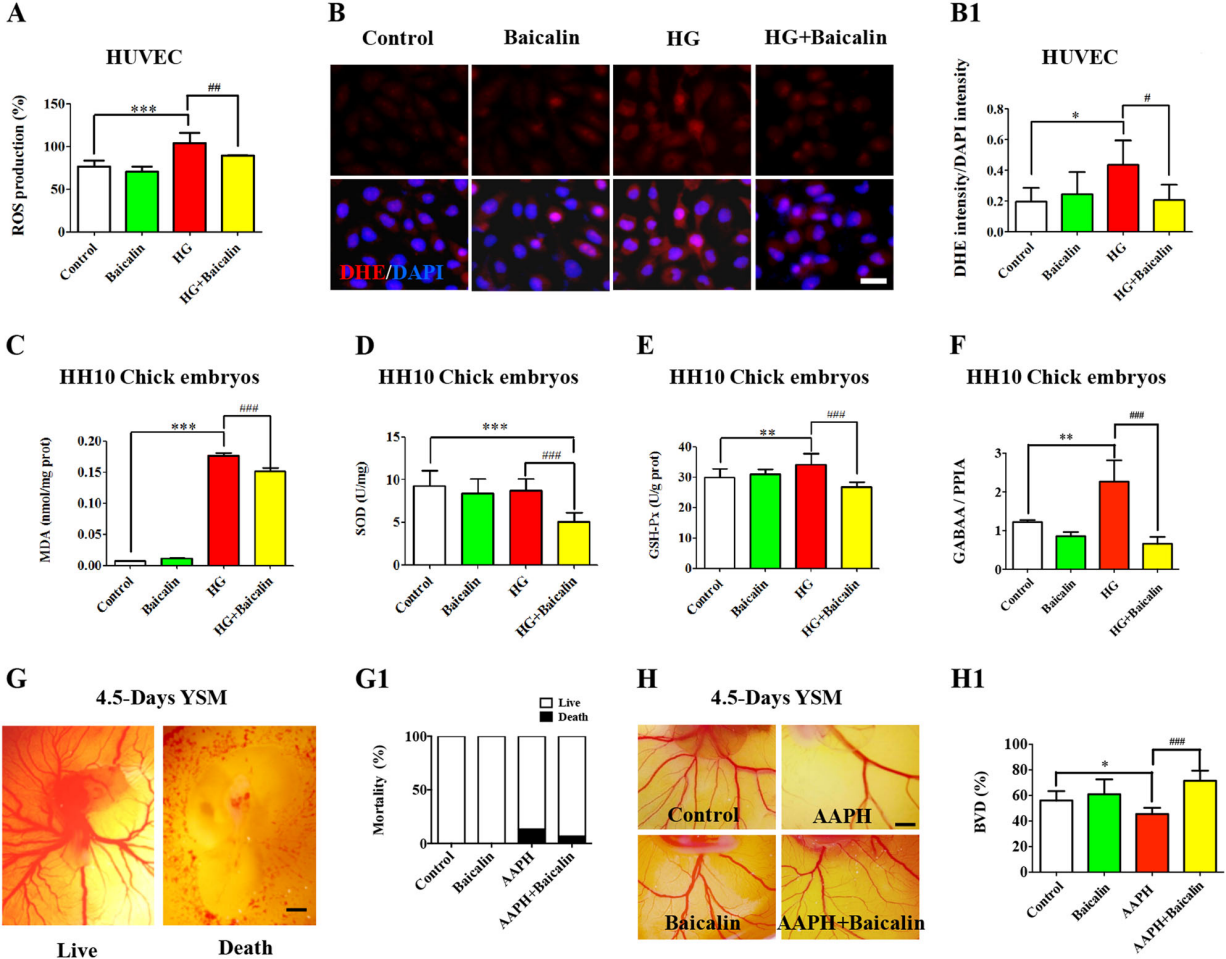
The redox homeostasis assessment in the absence/presence of HG and Baicalin. **a** Bar chart showing the comparisons of ROS production among 48-h incubated HUVECs from control, 6 μM Baicalin, 50 mM glucose and 6 μM Baicalin+50 mM glucose group. **b** Intracellular ROS production was determined using DHE fluorescent staining on the incubated HUVECs from control, 6 μM Baicalin, 50 mM glucose and 6 μM Baicalin+50 mM glucose group. **b1** Bar chart showing the ratio comparisons of DHE^+^ cells and total DAPI^+^ cells among each group. **c**–**e** Bar charts showing the comparisons of enzyme activities of SOD (**c**), MDA (**d**) and GSH-Px (**e**) among 48-h incubated HUVECs from control, 6 μM Baicalin, 50 mM glucose and 6 μM Baicalin+ 50mM glucose group. **f** Quantitative PCR data showing the expressions of GABAA in chick embryos and HUVECs exposed to either simple saline (control) or HG or/and Baicalin. **g**–**g1** The representative images were photographed from 4.5-day YSM exposed to either sample saline (control, left) or 3.75 mg/ml AAPH (right) (**g**), and the quantitatively analyzed embryo survival rates were shown in (**g1**). **h**–**h1** The representative images were photographed from 4.5-day YSM exposed to sample saline (control), 3.75 mg/ml AAPH, 6 μM Baicalin, and 6 μM Baicalin+3.75 mg/ml AAPH (**h**), and the quantitatively analyzed BDV (blood vessel density) was shown in (**h1**). *p<0.05 compared with control group; **p<0.01 compared with control group; ***p<0.001 compared with control group; ^##^p<0.01 compared with HG group; ^###^p<0.001 compared with HG group. Scale bars = 20 µm in (**b**) and 1000 µm in (**f**, **g**)

The obviously excessive ROS production was discovered in the presence of 50 mM glucose in HUVECs in vitro culture (*p* < 0.001, *n* = 5 in each group), but the addition of 6 μM Baicalin returned ROS levels back to normal (*p* < 0.01 compared with HG group, Fig. 6a). Staining with DHE, the cell-permeable fluorescent redox indicator of superoxide, demonstrated that 50 mM glucose administration in HUVECs could significantly increase the level of superoxide, but cotreatment with 6 μM Baicalin and 50 mM glucose could restore the level of superoxide (*p* < 0.05, Fig. 6b-b1). Additionally, the activities of SOD, MDA and GSH-Px were measured in HH10 chick embryos exposed to either Baicalin, glucose or in combination. The results demonstrated that MDA activity was distinctly elevated in the presence of 50 mM glucose (*p* < 0.001, Fig. 6c), but dropped sharply after addition of 6 μM Baicalin (*p *< 0.001 compared with HG group, *n* = 3 in each group). There were no significant alterations to SOD activities when the embryos were exposed to HG (*p* > 0.05, *n* = 8, Fig. 6d), but dropped significantly when treated with HG+Baicalin (*p* < 0.001, *n* = 8, Fig. 6d). GSH-Px activity was elevated in the presence of 50 mM glucose (*p* < 0.01 Fig. 6e), but the enzyme activities were decreased following addition of 6 μM Baicalin in the presence of 50 mM glucose (*p* < 0.001 compared with HG group, *n* > 6 in each group, Fig. 6e). MQAE staining for intracellular chloride ion concentration showed that there more fluorescent spots in the presence of HG, but were partially recovered after the addition of 6 μM Baicalin in HUVECs (Fig. S3, yellow arrows). Quantitative PCR data showed that the expressions of GABAA were up-regulated in the presence of HG (*p* < 0.01), but were partially recovered after addition of 6 μM Baicalin in chick embryos (*p* < 0.001 compared with HG group, *n* = 3, Fig. 6f).

Next, the environment of excessive ROS level was induced by the application of AAPH, a ROS generator, in chick YSM. AAPH (3.75 mg/ml) treatment resulted in the deaths of 13.40% of chick embryos as shown in Fig. 6g-g1, but only 6.67% chick embryo deaths were found on addition of 6 μM Baicalin after AAPH exposure (*n* = 30 embryos in each group). AAPH administration could significantly inhibit angiogenesis in YSM (*p *< 0.05), whereas a lesser effect was obtained following combined application of AAPH and Baicalin (*p* < 0.001, *n* > 7 in each group, Fig. 6h-h1), suggesting that oxidative stress could be one of the mechanisms underlying this protective effect on hyperglycemia-inhibited angiogenesis.

### Baicalin administration redressed the hyperglycemia-induced unbalanced secondary effect of autophagy on heart tube formation

**Fig. 7:**
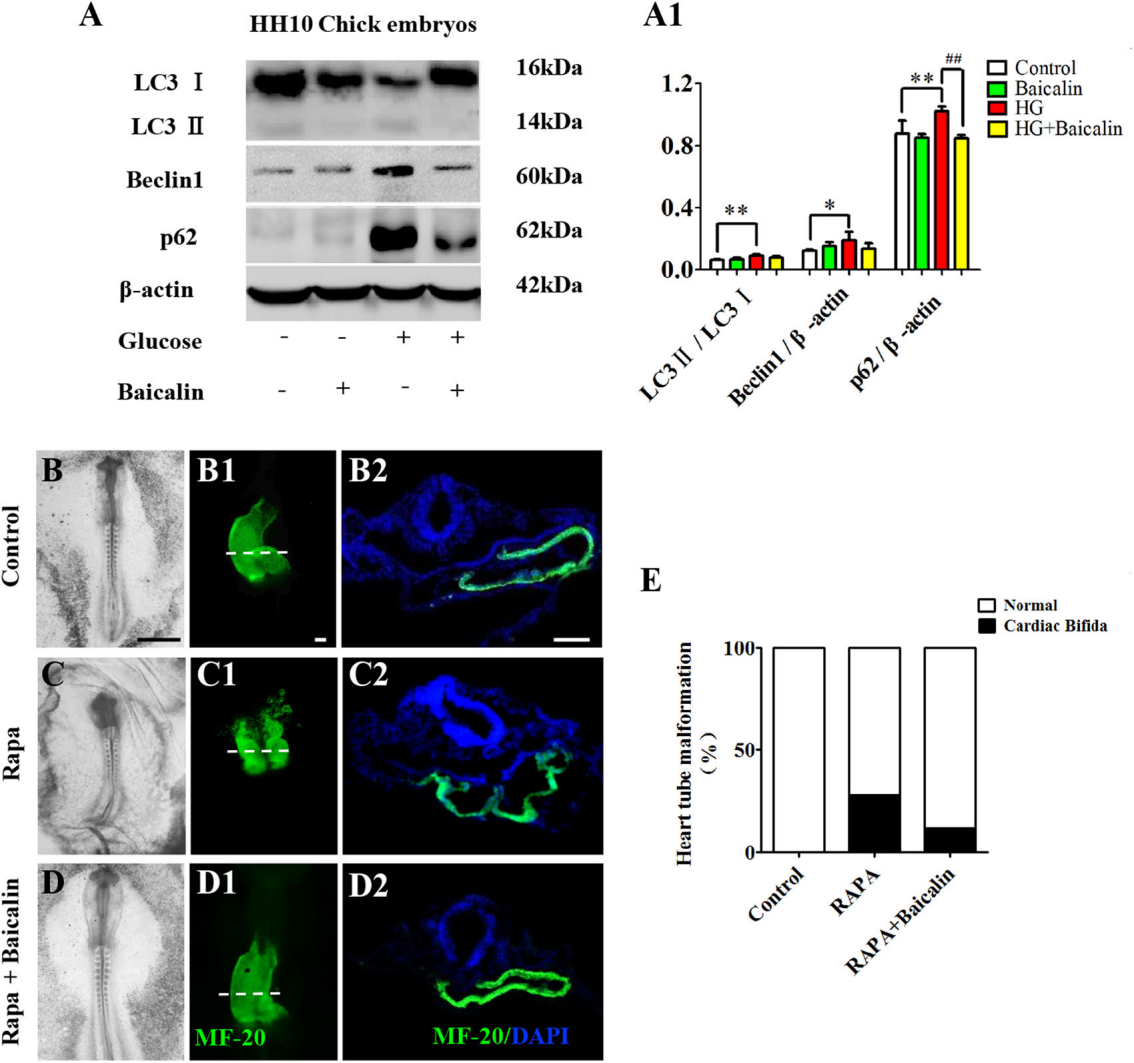
The assessment of autophagy in gastrula chick embryos and the incubated HUVECs in the absence/presence of Baicalin. **a**–**a1** Western blot data showing the expressions of LC3I, LC3II, p62 and Beclin1 in the opaca area of HH10 chick embryos from control, 6 μM Baicalin, 50 mM glucose and 6 μM Baicalin+50 mM glucose group (**a**), which was quantitatively analyzed in (**a1**). **b**–**d** The representative bright-field images of HH10 chick embryos exposed to sample saline (control) (**b**), Rapa (**c**) or Rapa+Baicalin (**d**). **b1**–**d1** The MF-20 immunofluorescent staining was implemented in HH10 chick embryos exposed to sample saline (control) (**b1**), Rapa (**c1**) or Rapa+Baicalin (**d1**). **b2**–**d2** The transverse sections at the levels indicated by dotted lines in (**b1**–**d1**), respectively. **e** Bar chart showing the comparison of cardiac bifida incidences in HH10 chick embryos exposed to sample saline (control), Rapa or Rapa+Baicalin. *p<0.05 compared with control group; **p<0.01 compared with control group; ^##^p<0.01 compared with HG group. Scale bars = 100 µm in (**b**–**d**), 10 µm in (**b1**–**d1**) and 100 µm in (**g**)

The western blot data showed that the ratio of LC3II/LC3I, Beclin1 and p62 were highly expressed in the presence of 50 mM glucose (*p* < 0.05, *p* < 0.01), and that the HG-enhanced expressions were suppressed in HH10 chick embryos by the administration of 6 μM Baicalin (*p* < 0.01, *n* = 3, Fig. 7a-a1). When Rapa was used to induce autophagy in early gastrula chick embryos, 28% cardiac bifida mortality of chick embryos was found (*n* = 25 in each group, Fig. 7b–e) but this was reduced to 12% when 6 μM Baicalin was added.

**Fig. 8:**
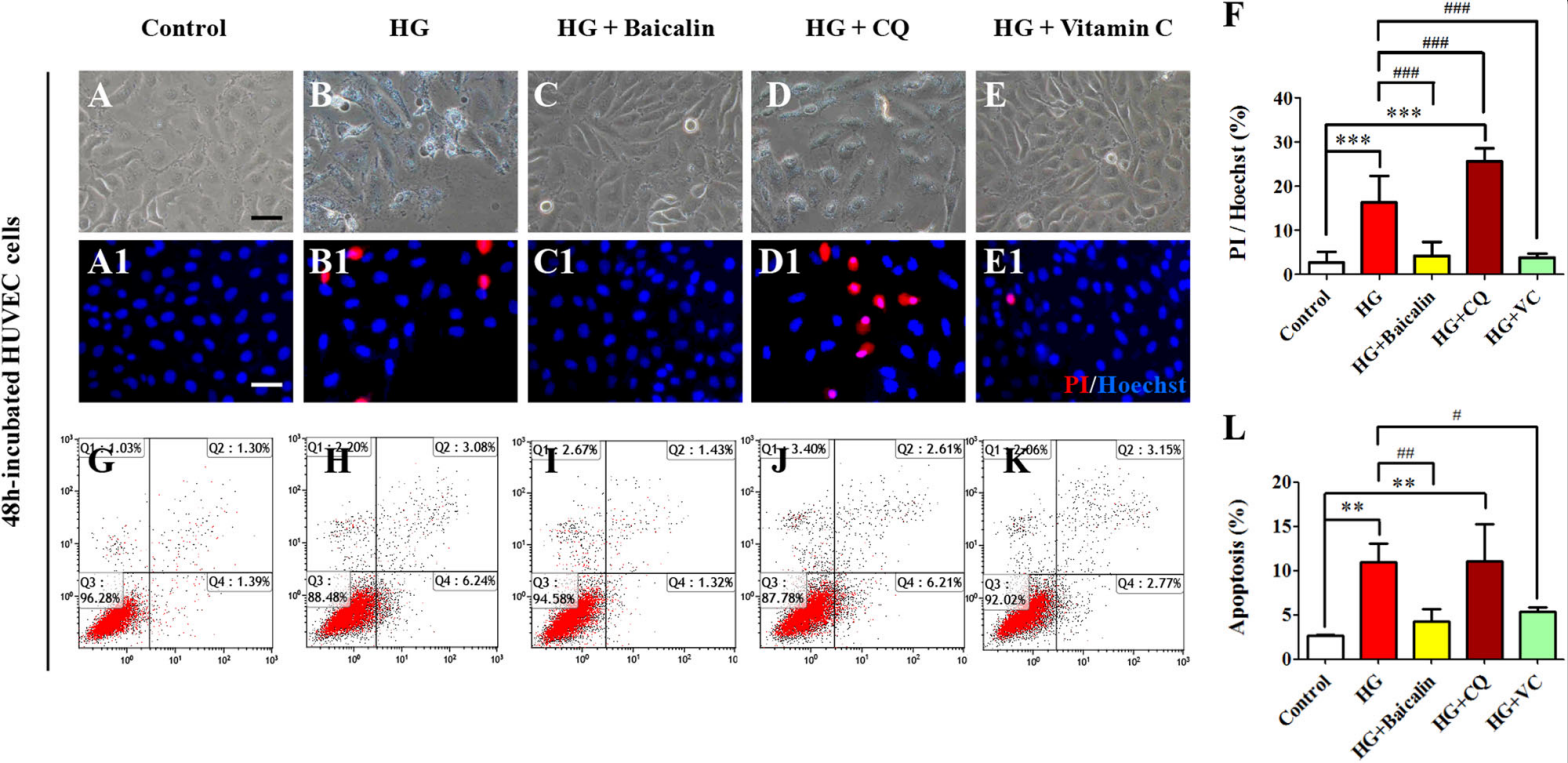
The apoptosis assessment of HUVECs in the absence/presence of HG and Baicalin, chloroquine or Vitamin C. **a**–**e** The representative bright-field images of 48-h cultured HUVECs from control (**a**), 50 mM glucose (**b**), 6 μM Baicalin+50 mM glucose (**c**), 10 μM chloroquine (CQ)+50 mM glucose (**d**) and 40 μM Vitamin C+50 mM glucose (**e**) group. **a1**–**e1** The fluorescent staining of PI and Hoechst was implemented on 48-h cultured HUVECs as in (**a**–**e**), respectively. **f** Bar chart showing the ratio comparison between PI^+^ and Hoechst^+^ HUVECs in each group. **g**–**k** The PI flow cytometric assay was implemented in mentioned-above groups. **l** The bar chart showing the comparison of the values of Q2 (late apoptosis)+Q4 (early apoptosis) in the cultured HUVECs among mentioned-above groups. **p<0.01 compared with control group; ***p<0.001 compared with control group; ^#^p<0.05 compared with HG group; ^##^p<0.01 compared with HG group; ^###^p<0.001 compared with HG group. Scale bars = 100 µm in (**a**–**e**) and (**a1**–**e1**)

To distinguish whether or not there are different roles of oxidative stress and autophagy in hyperglycemia-induced cardiovascular malformation, PI^+^ cell numbers were determined and PI flow cytometric assays conducted in HUVECs in the presence of HG alone and together with either Baicalin, Chloroquine (CQ) or Vitamin C (VC, Fig. 8). The results indicate that HG significantly elevated cell PI^+^ cell numbers compared with control group (*p* < 0.001, *n* = 3 in each group. Fig. 8a-b1,f); the addition of either 6 μM Baicalin or VC suppressed this elevation (*p* < 0.001 compared with HG group, *n* = 3. Fig. 8c-c1,e-e1,f). However, the addition of CQ did not reduce the HG-induced increase of cell PI^+^ cell numbers but were even increased further (*p* > 0.05 compared with HG group, Fig. 8d-d1,f). The flow cytometry data showed that 50 mM glucose could significantly enhance HUVEC apoptosis (*p* < 0.01, *n* = 3), and the increased apoptosis induced by HG was dramatically suppressed by the addition of 6 μM Baicalin or VC (*p* < 0.01, *p* < 0.05 compared with HG group, *n* = 3, Fig. 8h-i,k-l). However, addition of CQ failed to reduce the HG-induced apoptosis (*p* < 0.01 compared with control group, Fig. 8j,l) and PI^+^ cell numbers were again increased (*p* > 0.05 compared with HG group, Fig. 8h,j,l).

### Baicalin administration in pregnant mice could partially suppress STZ-induced hyperglycemia

**Fig. 9:**
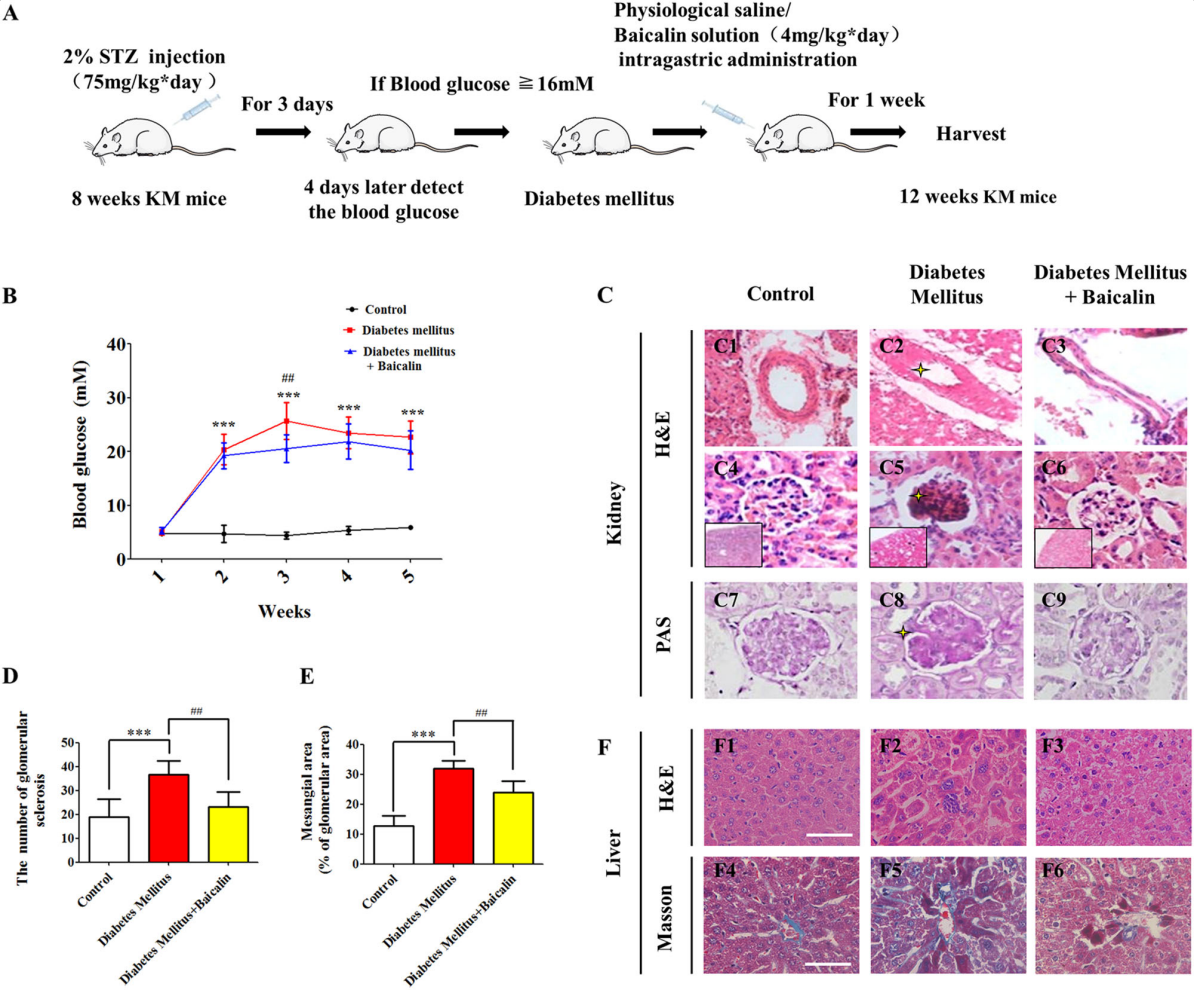
The blood glucose and histological alterations in STZ-induced diabetes mouse models following administration of Baicalin. **a** The sketches illustrating the mouse model of diabetes mellitus using STZ injection and Baicalin administration through intra-gastric administration and so on. **b** The blood glucose level in the treated mice was detected in control, diabetes mellitus and diabetes mellitus+Baicalin groups at weeks 1–5. **c** The representative H&E- and PAS-stained transverse sections of mouse kidneys from above three groups. **d**–**e** The bar charts showing the number comparison of glomerular sclerosis (**d**) and among control, diabetes mellitus, and diabetes mellitus+Baicalin groups. **f** The representative H&E (upper) or Masson (lower)-stained transverse sections of mouse livers from above three groups. ***p<0.001 compared with control group; ^##^p<0.01 compared with diabetes mellitus group. Scale bars = 100 µm in (**c**, **f**)

In order to examine whether the protective effect of Baicalin is a consequence of modulating maternal blood levels, pregnant STZ-induced diabetes mellitus mice were treated as shown in Fig. 9a. The blood glucose level was found to be significantly increased after 3 days of STZ injection, compared with the controls (second week: *p *< 0.001, *n* = 25, Fig. 9b). Diabetic mice were then randomly divided into two groups and one group was treated with Baicalin for 10 days. Compared to the diabetic controls, the blood glucose levels in the the group receiving Baicalin by intra-gastric administration were significantly lower after 3 weeks (*p* < 0.01 compared with the diabetes mellitus group, *n* = 11, 12, Fig. 9b). However, there was no significant difference at the 4–5-week stage. The histologies of kidneys and livers were then examined on the transverse sections. In comparison to the kidney control, endothelium damage was observed (Fig. 9c2), together with glomerular sclerosis (Fig. 9c5), hyperplasic glomeruli mesangial cells (Fig. 9c5), thickening of glomerular basement membrane (Fig. 9c8) as shown by stars, but such kidney structure damage was much less apparent in the mice receiving Baicalin (Fig. 9c3,c6,c9). Increases of glomerular sclerosis (diabetes mellitus group compared with control group: *p* < 0.001, Baicalin administration group compared with diabetes mellitus group: *p *< 0.01, Fig. 9d) and mesangial area (diabetes mellitus group compared with control group: *p* < 0.001, Baicalin administration group compared with diabetes mellitus group: *p* < 0.01, Fig. 9e) showed that diabetes was suppressed by Baicalin. Likewise, the degeneration and necrosis of hepatocytes in the transverse section of diabetes mellitus mouse liver compared to control was dramatically improved after Baicalin administration (Fig. 9f). These protective effects on kidney and liver might contribute to lowering elevated blood glucose levels in diabetic mice.

## Discussion

Baicalin is present as a glycoside in *Scutellaria baicalensis* Georgi, and is metabolized to its aglycone, Baicalein, by the action of intestinal β-glucuronidase in the intestine^33^. Significantly, Baicalin can be reformed into Baicalein, which is its previous metabolism, in many tissues. Both Baicalin and Baicalein can be detected in blood after absorption in the intestine^34^. The roots of *Scutellaria baicalensis* Georgi have been extensively used as traditional medicines in many East Asian countries to reduce inflammation^35^. Earlier studies have shown Baicalin and Baicalein to possess wide-ranging biological and pharmacological functions, including anti-inflammation, anti-cancer and anti-pruritic effects^36–38^. Most experiments have been carried out in vitro only, which simplifies the scientific protocols but makes extrapolation to the in vivo situation difficult, if not impossible. Therefore, it is crucial to conduct well-designed in vivo experiments to investigate these compounds’ pharmacological and toxicological effects. As one of the first systems to develop in early pregnancy, the cardiovascular system is undoubtedly vulnerable to diabetes mellitus-induced hyperglycemia exposure. Dysplasia of the cardiovascular system during cardiogenesis and angiogenesis leads to constantly impaired fetal development or death. Therefore, in this study, the focus has been on whether Baicalin administration could improve hyperglycemia-induced cardiovascular dysplasia.

Most of the experiments in this study have been carried out using chick embryos. The advantage of this model is that chick embryos can grow outside of uterus. They can thus be easily manipulated, and can be especially useful for the study of effects on early embryo development in EC culture or angiogenesis using YSM and CAM. The chick embryo model allows direct monitoring of the development of the embryos at any stage following the experimental manipulation in either EC culture or windowed eggshell^39^. Song et al.^40^ reported the potential embryotoxicity of Baicalin when they evaluated the safety of Shuanghuanglian injection powder, in which Baicalin is the main component, through placental barriers of rats. The placental permeability of Baicalin undoubtedly enables it to adversely affect embryonal development after early embryo exposure.

For this reason, embryotoxic experiments were initially conducted. Using the EC culture approach, an optimal concentration of Baicalin was selected for administration in subsequent experiments that did not influence chick embryo development (Fig. 1). At the same time, hyperglycemia-inhibited embryo development was significantly attenuated (Fig. 2). In the process of heart tube formation, Baicalin administration could distinctly reduce the risk of hyperglycemia-induced cardiac bifida by reversing the expressions of the crucial genes related to heart tube development (Fig. 3). In evaluating the effect of Baicalin on vasculature development, the blood island formation model (vasculogenesis) in area opaca and the CAM model (angiogenesis), which include early stage of blood islands/primary vessel plexus (Fig. 4), were employed along with the later stage of angiogenesis (Fig. S2). Obviously, the inhibitive effect of hyperglycemia on blood island formation was greatly reduced in the presence of Baicalin (Fig. 4), while hyperglycemia-induced angiodysplasia on CAM was also minimized by Baicalin administration (Fig. S2). Taken together, these data suggest that Baicalin administration has a positive impact on hyperglycemia-induced cardiovascular dysplasia during early embryo development.

Hyperglycemic conditions can increase both apoptotic cell death and intracellular ROS generation^41,42^. Therefore, an investigation of whether apoptosis is a key link for Baicalin against hyperglycemia-induced cardiovascular malformation was carried out. Cell apoptosis induced by hyperglycemic conditions was dramatically reversed by Baicalin administration (Fig. 5). SOD, GSH-Px and MDA were all decreased after addition of Baicalin to HG-treated chick embryos (Fig. 6c-e). This suggested that Baicalin might also play an antioxidant role in chick embryos, but not through activating SOD and GSH-Px. Cell size was reduced after HG exposure (Fig. 5i). The loss of cell volume or cell shrinkage is a known morphological hallmark of apoptosis^43,44^. Activation of the volume-sensitive outwardly rectifying Cl(−) channel is involved in this process^45^. Detection of intracellular chloride ion showed a reduction of Cl(−) in the presence of HG and that can be reversed by the addition of Baicalin (Fig. S3). GABAA, an important Cl(−) channel, is sensitive to ROS and recent studies have demonstrated that Baicalin is acting through GABAA^5^. The changes of GABAA shown here (Fig. 6f) suggested that the antioxidant function of Baicalin is caused by suppression of outwardly rectifying Cl(−) in the HG microenvironment. Meanwhile, both the in vivo and in vitro experiments imply that the hyperglycemia-induced angiodysplasia could be partially rectified through suppressing excessive ROS production induced by HG (Fig. 6g,h). These observations further confirm the antioxidant bioactivity of Baicalin^8,46^.

Autophagy has been shown to be involved in high glucose-induced heart tube malformation^22^. As in earlier reports^47,48^ high glucose was observed to increase the expression of p62, suggesting that autophagy flux is inhibited. However, after Baicalin was added, the expression of the hyperglycemia-enhanced autophagy gene decreased and cardiac bifida induced by Rapa-induced excessive autophagy disappeared (Fig. 7). These results suggest that Baicalin might increase the ubiquitin of p62 and hence accelerate autophagy flux. These processes could facilitate the cleanup of damaged organelles. In order to examine this further and determine correlations between oxidative stress and autophagy, the effect of Baicalin on cell apoptosis was studied following addition of either Baicalin or CQ (blocking autophagy) or Vitamin C (antioxidant) under HG conditions. As Baicalin administration attenuated cell apoptosis, suppressing ROS excessive production with Vitamin C could reduce the cell apoptosis, but attempts to block autophagy with CQ had no effect (Fig. 8). This suggests that massive ROS-induced cell autophagy plays an important role in cell apoptosis and that autophagy should exert a protective function on cell survival.

In consideration of maternal factors during application of Baicalin, STZ-induced diabetes mellitus mice were administered the compound. The results showed that Baicalin administration could attenuate diabetes mellitus-induced damage of kidney and liver to some extent; and successfully reduce the blood glucose levels of diabetic mice induced by STZ (Fig. 9) over the following weeks.

**Fig. 10:**
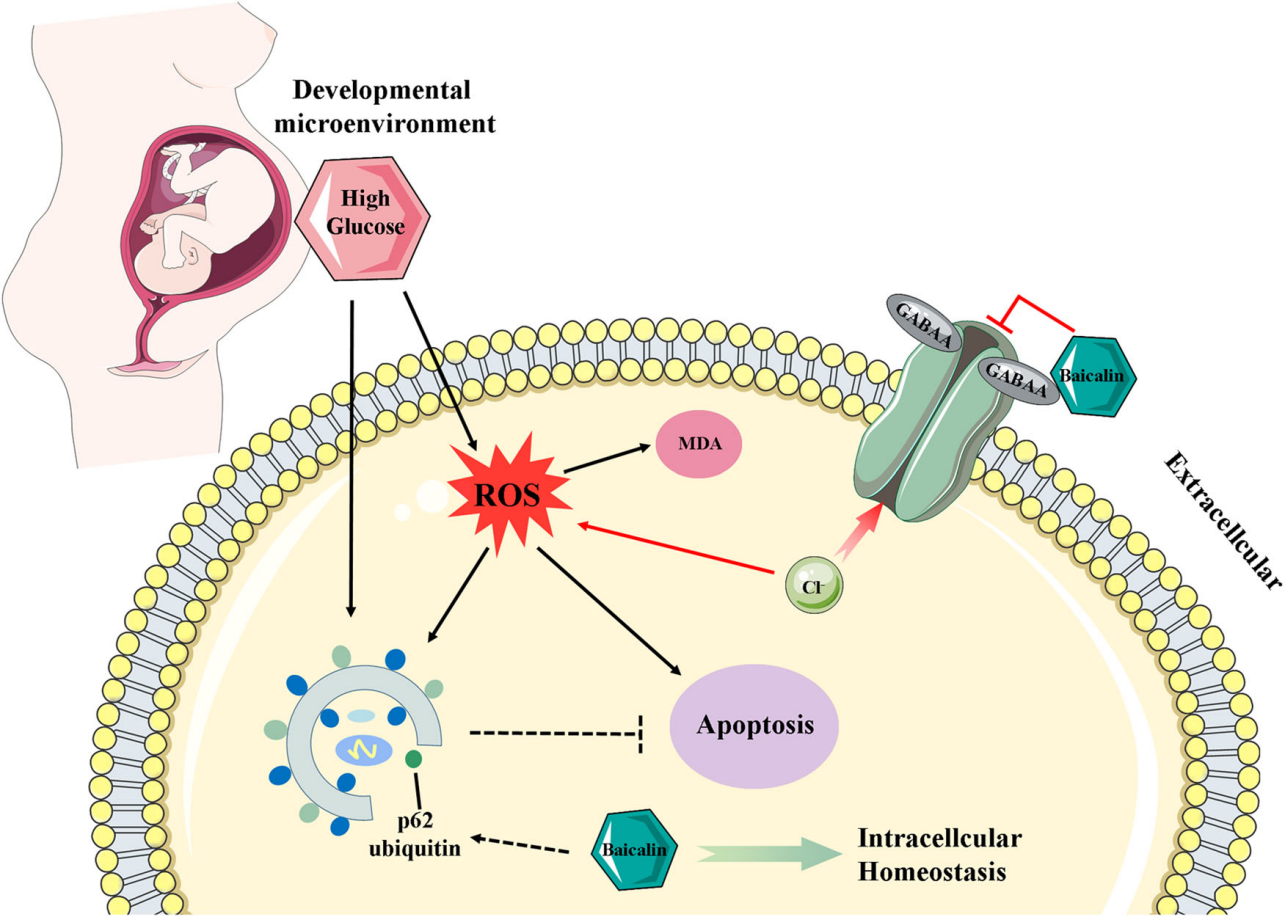
Model depicting how Baicalin administration improves HG-induced malformation of embryonic cardiovascular system. Being one of the earliest developmental systems, the cardiovascular system is undoubtedly vulnerable to diabetes mellitus-induced hyperglycemia exposure in early pregnancy. Dysplasia of the cardiovascular system during cardiogenesis and angiogenesis leads to constantly impaired fetal development or death. However, there is no effective drug for the protection of embryonic development of gestational diabetes mellitus. Both Glucose and Baicalin could transfer through placental barriers and then expose to the embryo. Therefore, in this study, the focus has been on whether Baicalin administration could improve hyperglycemia-induced cardiovascular dysplasia in chick embryo. We found that Baicalin protects cardiovascular formation from HG-induced damage through two mechanisms: (1) reducing hyperglycemia-enhanced excessive ROS generation might involve suppression of outwardly rectifying Cl(−) and subsequent cell apoptosis; (2) promoting autophagy flow through regulating the p62 to maintain the intracellular homeostasis. Therefore, Baicalin could be a potential candidate drug for gestational diabetes mellitus women

In summary (Fig. 10), Baicalin administration in mice could partially confront maternal hyperglycemia. Meanwhile, Baicalin could cross the placental barrier to protect cardiovascular formation from HG-induced damage. The exact mechanisms involved in reducing hyperglycemia-enhanced excessive ROS generation might involve suppression of outwardly rectifying Cl(−); unbalanced autophagy through regulating the p62 and subsequent cell apoptosis mainly induced by ROS. Therefore, embryonic angiodysplasia and heart tube malformation induced by hyperglycemia could be partially reduced through the intracellular homeostasis resulting from Baicalin administration, and Baicalin could be a potential candidate drug for gestational diabetes mellitus women. Further experimentations are required to investigate the therapeutic potential in treatment of diabetes and to determine the precise molecular mechanisms involved.

## Electronic supplementary material

Supplementary Fig 1

Supplementary Fig 2

Supplementary Fig 3

Supplementary Results (We revised the Supplementary Results since there were several spell errors. We uploded it in the attachments.)

Supplementary Figure legends
