# Supplementary figures and images for: Baicalin administration attenuates hyperglycemia-induced malformation of cardiovascular system
Source: Cell Death Dis. 2018 Feb 14;9(2):234. doi: 10.1038/s41419-018-0318-2 (PMC5833405; doi:10.1038/s41419-018-0318-2)

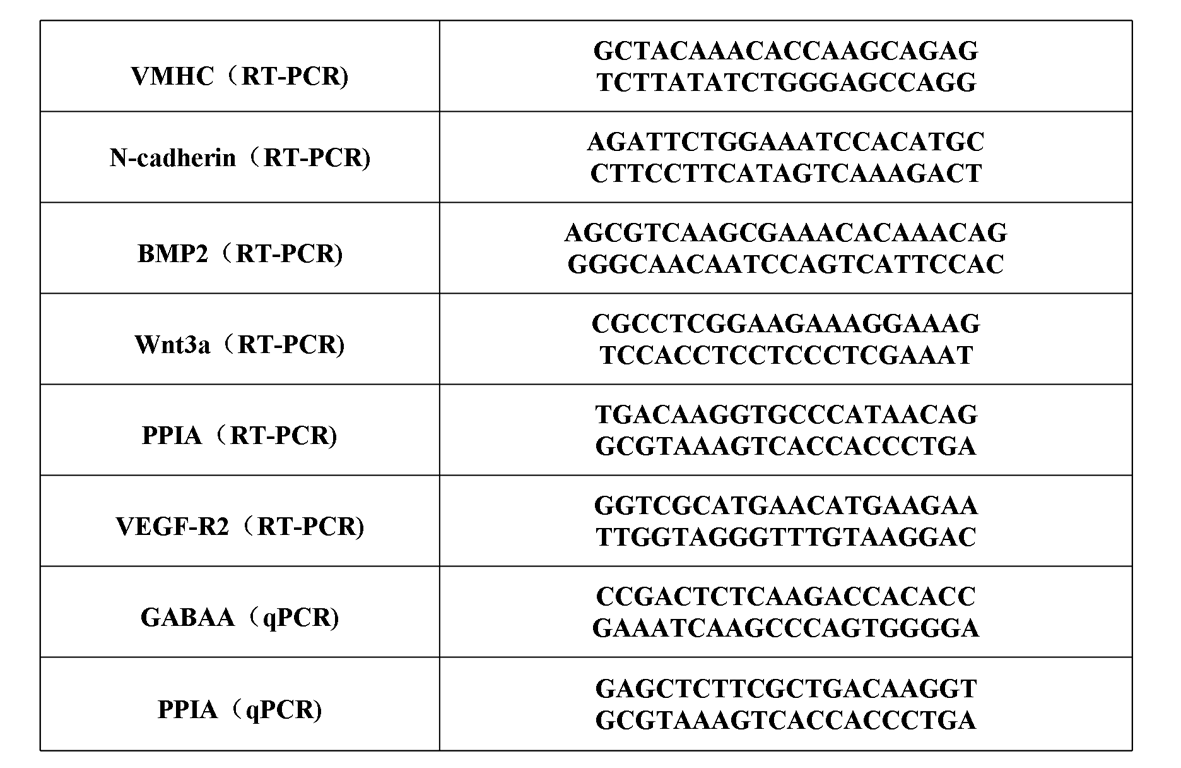

Supplement: Supplementary file 1 — Supplementary Fig 1 [file 41419_2018_318_MOESM1_ESM.tif]

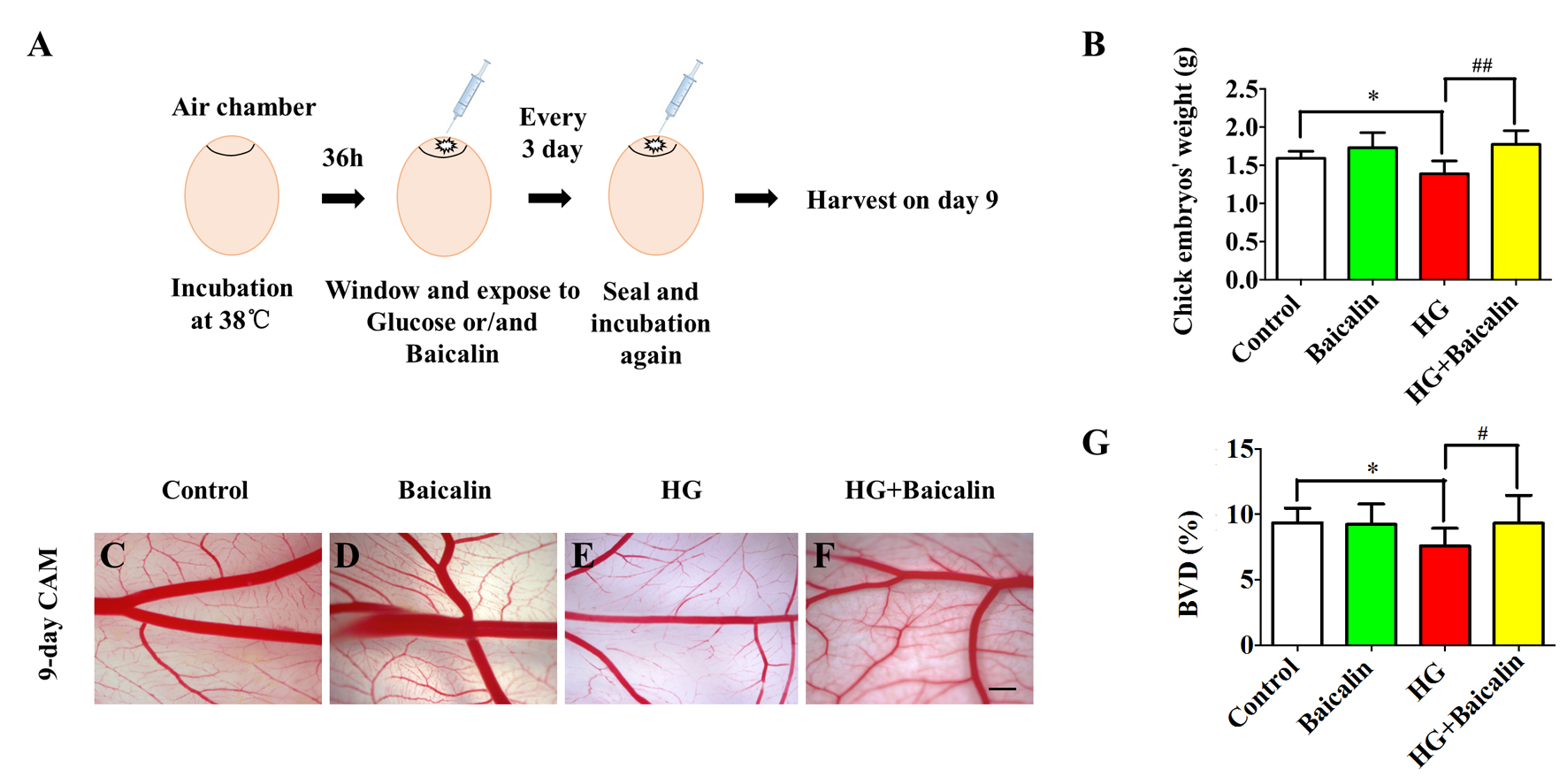

Supplement: Supplementary file 2 — Supplementary Fig 2 [file 41419_2018_318_MOESM2_ESM.tif]

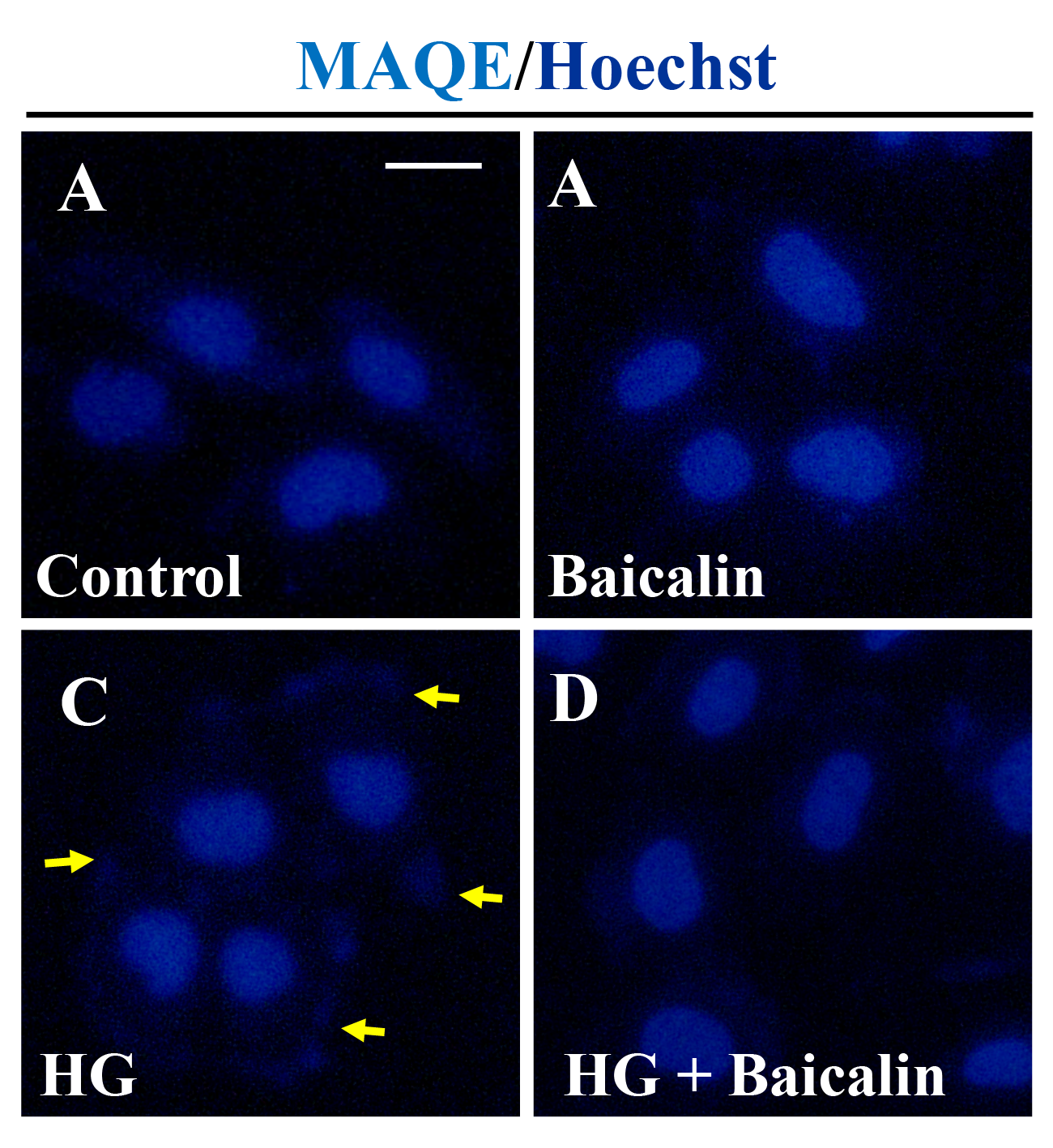

Supplement: Supplementary file 3 — Supplementary Fig 3 [file 41419_2018_318_MOESM3_ESM.tif]
