# Supplementary material for: Baicalin administration attenuates hyperglycemia-induced malformation of cardiovascular system
Source: Cell Death Dis. 2018 Feb 14;9(2):234. doi: 10.1038/s41419-018-0318-2 (PMC5833405; doi:10.1038/s41419-018-0318-2)
Supplement: Supplementary file 5 — Supplementary Figure legends [file 41419_2018_318_MOESM5_ESM.docx]

**Supplementary Results**

**Fig. 1:**

**B1:** Control: 0.58 ± 0.05mm; 3μM baicalin: 0.57 ± 0.07mm; 6μM baicalin: 0.59± 0.04mm; 12 μM baicalin: 0.48 ± 0.04mm; 24μM baicalin: 0.39 ± 0.04mm. N>6 embryos in each group.

**B2:** Control: 4.06 ± 0.13mm; 3μM baicalin: 4.02 ± 0.18mm; 6μM baicalin: 3.95 ± 0.29mm; 12 μM baicalin: 3.47 ± 0.29mm; 24μM baicalin: 3.08 ± 0.38mm. N>6 embryos in each group.

**B3:** Control: 4.28 ± 0.09mm; 3μM baicalin: 4.15 ± 0.16mm; 6 μM baicalin:4.27 ± 0.09mm; 12μM baicalin: 3.60 ± 0.29 mm; 24 μM baicalin: 3.79 ± 0.16mm. N>6 embryos in each group.

**B4:** Control: 13.80 ± 0.60; 3μM baicalin: 14.00 ± 0.77; 6μM baicalin:13.60 ± 0.67; 12μM baicalin: 10.90 ± 1.70; 24μM baicalin: 9.20 ± 1.47. N>6 embryos in each group.

**C1:** Control: 0%; 3μM baicalin: 0%; 6μM baicalin: 0%; 12μM baicalin: 33%; 24μM baicalin: 50%. N>6 embryos in each group.

**Fig. 2:**

**A1:** Control: 0.58 ± 0.05mm; 0μM baicalin + 50Mm glucose: 0.38 ± 0.44mm; 3μM baicalin + 50Mm glucose: 0.48 ± 0.04mm; 6μM baicalin + 50Mm glucose: 0.46 ± 0.07mm. N>6 embryos in each group.

**A2:** Control: 4.06 ± 0.13mm; 0μM: 3.30 ± 0.35mm; 3μM: 3.52 ± 0.20mm; 6μM: 3.95 ± 0.08mm. N>6 embryos in each group.

**A3:** Control: 4.28 ± 0.09mm; 0μM: 3.35 ± 0.17mm; 3μM: 3.79 ± 0.25mm; 6μM: 4.26 ± 0.16mm. N>6 embryos in each group.

**A4:** Control: 13.80 ± 0.60; 0μM: 9.20 ± 1.17; 3μM: 10.00 ± 1.41; 6μM: 12.90 ± 0.70. N>6 embryos in each group.

**B:** Control: 0%; HG+0μM baicalin: 50%; HG+3μM baicalin: 50%; HG+6μM baicalin: 20%. N>6 embryos in each group.

**Fig. 3:**

**E:** Heart tube malformation: Control: 0%; Baicalin: 0%; HG: 32%; HG+Baicalin: 16%. N=25 in each group.

**F1:** VMHC/PPIA: Control: 0.33 ± 0.0280; Baicalin: 0.30 ± 0.0293; HG: 0.24 ± 0.0147; HG+Baicalin: 0.36± 0.0187. N>3.

N-Cadherin / PPIA: Control: 0.36 ± 0.0288; Baicalin: 0.34 ± 0.0089; HG: 0.15 ± 0.0155; HG+Baicalin: 0.25 ± 0.0391. N>3.

BMP2 / PPIA: Control: 1.02 ± 0.0177; Baicalin: 0.95 ± 0.0728; HG: 0.50 ± 0.0469; HG+Baicalin: 0.69 ± 0.0390. N>3.

Wnt3a / PPIA: Control: 0.22 ± 0.0683; Baicalin: 0.14 ± 0.0243; HG: 0.05 ± 0.0144; HG+Baicalin: 0.12 ± 0.0298. N>3.

**G:** GATA4/β- actin: Control: 0.11 ± 0.0038; Baicalin: 0.11 ± 0.0069; HG: 0.09 ± 0.0058; HG+Baicalin: 0.12± 0.0015. N>3.

**Fig. 4:**

**F:** BVD(%): Control: 20.61 ± 7.0017%; Baicalin: 22.69 ± 5.3238%; HG: 8.91 ± 1.0489%; HG+Baicalin: 16.85 ± 3.8554%. N=8 in each group.

**G:** VEGFR2/PPIA: Control: 0.66 ± 0.0893; Baicalin: 0.51 ± 0.0607; HG: 0.36 ± 0.1233; HG+Baicalin: 0.73 ± 0.1369. N>3.

**Fig. 5:**

**A:** c-Caspase3/β- actin : Control: 0.28 ± 0.0011; Baicalin: 0.29 ± 0.0179; HG: 0.33 ± 0.0085; HG+Baicalin: 0.30 ± 0.0045. N>3.

**B:** 6hour: Control: 1.00 ± 0.07; Baicalin: 1.04 ± 0.09; HG: 0.93 ± 0.13; HG+Baicalin: 0.94 ± 0.04.

12hour: Control: 1.00 ± 0.06; Baicalin: 0.97 ± 0.09; HG: 0.80 ± 0.03; HG+Baicalin: 0.91 ± 0.09.

24hour: Control: 1.00 ± 0.07; Baicalin: 1.01 ± 0.10; HG: 0.71 ± 0.06; HG+Baicalin: 0.88 ± 0.04.

36hour: Control: 1.00 ± 0.02; Baicalin: 1.00 ± 0.03; HG: 0.0.69 ± 0.04; HG+Baicalin: 0.96 ± 0.03.

48hour: Control: 1.12 ± 0.10; Baicalin: 1.21 ± 0.06; HG: 0.51 ± 0.02; HG+Baicalin: 1.18 ± 0.03.

N>4 in each group.

**C1:** Apotosis: Control: 4.49 ± 2.22%; Baicalin: 6.57 ± 2.50%; HG: 13.69 ± 2.83%; HG+Baicalin: 9.63 ± 3.13%. N=3 in each group.

**I:** PI/Hochest: Control: 3.36 ± 1.6068%; Baicalin: 2.39 ± 0.7012%; HG: 25.65 ± 2.4128%; HG+Baicalin: 3.12 ± 0.5646%. N=3 in each group.

**J:** Cell area: Control: 0.36 ± 0.12mm²; Baicalin: 0.35 ± 0.07 mm²; HG: 0.27 ± 0.10 mm²; HG+Baicalin: 0.38 ± 0.12 mm². N >18 in each group.

**Fig. 6:**

**A:** ROS production (%): Control: 76.60 ± 6.12%; Baicalin: 70.60 ± 5.24%; HG: 104.20 ± 10.42%; HG+Baicalin: 89.60 ± 0.49%. N=5 in each group.

**B1:** DHE intensity/DAPI intensity: Control: 0.20 ± 0.08; Baicalin: 0.24 ± 0.14; HG: 0.44 ± 0.14; HG+Baicalin: 0.21 ± 0.09. N>6 in each group.

**C:** SOD: Control: 9.27 ± 1.70U/mg; Baicalin: 8.34 ± 1.65U/mg; HG: 8.74 ± 1.24U/mg; HG+Baicalin: 5.11 ± 0.95U/mg. N=8 in each group.

**D:** MDA: Control: 0.01 ± 0.00μmol/mg prot; Baicalin: 0.01 ± 0.00μmol/mg prot; HG: 0.18 ± 0.00μmol/mg prot; HG+Baicalin: 0.15 ± 0.00μmol/mg prot. N=3 in each group.

**G:** Mortality: Control: 0%; Baicalin: 0%; AAPH: 32%; AAPH +Baicalin:16%. N=30 embryos in each group.

**H1:** YSM BVD(%): Control: 56.14± 7.3379%; Baicalin: 60.99 ± 11.6163%; AAPH: 45.51 ± 4.8439%; AAPH +Baicalin: 71.57 ± 7.9248%. N>7 in each group.

**Fig. 7:**

**A1:** LC3II/LC3I: A: Control: 0.06 ± 0.0007; Baicalin: 0.07 ± 0.0101; HG: 0.09 ± 0.0087; HG+Baicalin: 0.08 ± 0.0090. N=3 in each group.

Beclin1/β-actin: A: Control: 0.12 ± 0.0059; Baicalin: 0.15 ± 0.0235; HG: 0.19 ± 0.0545; HG+Baicalin: 0.13 ± 0.0362. N=3 in each group.

P62/β-actin: A: Control: 0.88 ± 0.0851; Baicalin: 0.85 ± 0.0234; HG: 1.02 ± 0.0302; HG+Baicalin: 0.85 ± 0.0194. N=3 in each group.

**E:** Control: 0%; Baicalin: 0%; RAPA: 28%; RAPA+Baicalin: 12%; N=25 embryos in each group.

**Fig. 8:**

**F:** PI/Hochest(%): Control: 2.7 ±2.271%; HG: 16.3 ±5.510%; HG+Baicalin: 4.2 ± 2.920%; HG+CQ: 25.6 ±2.767%; HG+VC: 3.8 ±21.89%. N=3 in each group.

**L:** Apotosis (%): Control: 2.64 ± 0.0927%; HG: 10.96 ± 1.6853%; HG+Baicalin: 4.24 ± 1.1853%; HG+CQ: 11.04 ± 3.4237%; HG+VC: 5.41 ± 0.6316%. N=3 in each group.

**Fig. 9:**

**B:** Control: week 1: 4.77 ± 0.27mM; week 2: 4.75 ± 1.60mM; week 3: 4.38 ± 0.64 mM; week 4: 5.40 ± 0.74 mM; week 5: 5.85 ± 0.41 mM. N=6.

Diabetes Melitus: week 1: 5.20 ± 0.66mM; week 2: 20.38 ± 2.82mM; week 3: 25.67 ± 3.45 mM; week 4: 23.45 ± 2.94mM; week 5: 22.65 ± 3.07mM. N=11.

Diabetes Melitus+Baicalin: week 1: 5.30 ± 0.61mM; week 2: 19.23 ± 2.39 mM; week 3: 20.54 ± 2.54mM; week 4: 21.88 ± 3.29mM; week 5: 20.28 ± 3.53mM. N=12.

**D:** The number of glomerular sclerosis: Control: 19.00 ± 6.8069; Diabetes Melitus: 36.67 ± 5.2175; Diabetes Melitus+Baicalin: 23.17 ± 5.7276. N=6 in each group.

**E:** Mesangial area (% of glomerular area): Control: 12.79 ± 3.42%; Diabetes Melitus: 32.02 ± 2.48%; Diabetes Melitus+Baicalin: 23.92 ± 3.82%. N>5 in each group.

**Supplementary Fig. 2.**

**B:** Control: 1.5 ± 0.11g; Baicalin: 1.70 ± 0.23g; HG: 1.47 ± 0.18g; HG+Baicalin: 1.89 ± 0.24g. N>10 embryos in each group.

**G:** BVD(%): A: Control: 9.35 ± 1.1149%; Baicalin: 9.23 ± 1.5669%; HG: 7.58 ± 1.3476%; HG+Baicalin: 9.32 ± 2.1318%. N>8 embryos in each group.
